# Description of a novel species of *Leclercia*, *Leclercia tamurae* sp. nov. and proposal of a novel genus *Silvania* gen. nov. containing two novel species *Silvania hatchlandensis* sp. nov. and *Silvania confinis* sp. nov. isolated from the rhizosphere of oak

**DOI:** 10.1186/s12866-022-02711-x

**Published:** 2022-12-02

**Authors:** Daniel Maddock, Dawn Arnold, Sandra Denman, Carrie Brady

**Affiliations:** 1 Centre for Research in Bioscience, School of Applied Sciences, University of the West of England, Bristol, BS16 1QY UK; 2 Harper Adams University, Shropshire, Newport, UK; 3 Centre for Ecosystems, Society and Biosecurity, Forest Research, Farnham, UK

**Keywords:** Acute oak decline, Rhizosphere, *Leclercia*, Plant growth promoting bacteria, Plant growth-promoting traits, *Enterobacteriaceae*, *Silvania*

## Abstract

**Background:**

Acute Oak Decline (AOD) is a decline disease first reported on native oaks in the UK, but in recent years reports from further afield such as Europe and the Middle East, indicate that the distribution and host range is increasing at an alarming rate. The stem weeping symptoms of the disease partially develop due to polymicrobial-host interaction, caused by several members of the order Enterobacterales. While investigating the rhizosphere soil of AOD-unaffected trees, termed ‘healthy’ trees, and diseased oaks suffering from Acute Oak Decline (AOD), an enrichment method designed for enhanced recovery of Enterobacterales led to the recovery of several isolates that could not be classified as any existing species. These isolates showed a close relationship to the genus *Leclercia*, of which both species are of clinical importance, but the type species *Leclercia adecarboxylata* also displays plant growth-promoting properties in the rhizosphere.

**Results:**

Partial sequencing of four housekeeping genes revealed similarity to the genus *Leclercia* with varying degrees of relatedness. As such a complete polyphasic approach was used to determine the true taxonomic position of these isolates. This involved whole genome sequencing, phylogenomic analysis, phylogenetic analysis of both the 16S rRNA and four housekeeping gene sequences, combined with phenotypic testing and fatty acid analysis. Both the phylogenomic and phylogenetic analyses separated the isolates into four clusters, two of which were contained in the *Leclercia* clade. The remaining two clusters formed a separate lineage far removed from any currently defined species. Further investigation into the role of the isolates as plant growth-promoting bacteria as well as plant pathogens was investigated computationally, revealing a number of plant growth-promoting traits as well as virulence genes related to motility, adhesion and immune modulation.

**Conclusion:**

Based on the genotypic and phenotypic data presented here, these isolates could be differentiated from each other and their closest neighbours. As such we propose the description of *Leclercia tamurae* sp. nov. (type strain H6S3^T^ = LMG 32609^T^ = CCUG 76176^T^), *Silvania* gen. nov., *Silvania hatchlandensis* sp. nov. (type strain H19S6^T^ = LMG 32608^T^ = CCUG 76185^T^) and *Silvania confinis* sp. nov. (type strain H4N4^T^ = LMG 32607^T^ = CCUG 76175^T^). Due to their interesting protein annotations and alignments, these species warrant further investigation for their role in relation to plant health.

**Supplementary Information:**

The online version contains supplementary material available at 10.1186/s12866-022-02711-x.

## Background

In 1962 H. Leclerc, a key figure in the understanding of enteric bacteria, proposed the name *Escherichia adecarboxylata* for a novel bacterial species in the family *Enterobacteriaceae* [1]*.* The majority of strains originally isolated by Leclerc, were from food and notably produced a yellow pigment, when tested by IMViC the isolates resembled *Escherichia coli* [2]. The following 20 years proved tumultuous for *E. adecarboxylata* as it was first described as another synonym of *Enterobacter agglomerans* syn. *Erwinia herbicola* (now *Pantoea agglomerans*) [3]. This proposal was subsequently re-evaluated based on the separate studies by Brenner [4] and Farmer et al. [5] who used DNA-DNA hybridisation and biochemical assays, respectively, to identify the heterogenous nature of *E. agglomerans* [5]. Finally, following the biochemical and DNA hybridisation studies of 86 isolates from clinical, food and water samples and the environment, it was proposed to transfer *E. adecarboxylata* to a novel genus as *Leclercia adecarboxylata* [6].

Since its description in the late 1980s, literature on the genus *Leclercia* remained uncommon. However, publications relating to *L. adecarboxylata* infections and risks to human health have risen in recent years with multidrug resistant strains being isolated from both bovine samples and humans suffering from respiratory disease [7–9]. Infections are thought to arise from the environment, where *Leclercia* is a generalist and regularly isolated from soil and water [6, 7]. This suggestion was supported following a soft tissue infection caused by *L. adecarboxylata* on an injury gained while surfing [10]. However, in its normal environment, *Leclercia* may also play more beneficial roles to plants as members of the rhizosphere, where it has been repeatedly isolated and shown to exhibit plant growth-promoting qualities [11]. Most recently, a monospecific species, *Leclercia pneumoniae*, was described in the genus [12]. The strain, isolated from an infant with pneumonia and septicaemia at the Leipzig University Hospital, was shown to be a novel species through whole genome average nucleotide identity, phenotypic (MALDI-TOF and substrate utilisation) and phylogenomic comparison to *L. adecarboxylata* and other closely related species.

While investigating the role of the rhizosphere in the cause and development of Acute Oak Decline (AOD) in the present study, several potential novel species of *Leclercia*, identified by partial *gyrB* sequencing, were isolated from rhizosphere soil collected from Hatchlands Park, Guildford, UK. AOD is a decline disease that was first reported on native oak in the UK but is now seen to have a wider range of hosts and locations. AOD has recently been reported in Spain, Switzerland, Poland, Portugal, Latvia and Iran, with symptoms observed on other species of oak aside from *Quercus petraea* and *Quercus robur* [13–17]. The weeping stem lesions, which are characteristic symptoms of the disease have a polymicrobial cause in which *Brenneria goodwinii* and *Gibbsiella quercinecans* have been identified as the causative agents [18]. Decline diseases by definition have multiple predisposing, contributing and inciting factors that cause a healthy tree to spiral into decline and eventually death [19]. The model has recently been updated to include the role of the microbiome in predisposition, which includes the root microbiome and the bacteria they interact with in their rhizosphere [20]. The rhizosphere is a key feature of plant health via root function, being the first point of contact between soil and plants. Both plant growth-promoting bacteria, that mobilise nutrients and play antagonistic roles to pathogens, and phytopathogens themselves thrive in this area [21]. Recent studies have shown that distinct differences between the bacterial community composition of the rhizospheres associated with both healthy and diseased oak suffering from AOD can be observed [22]. These differences can affect oak health, for example by the association of ammonia-oxidising bacteria increasing nitrogen content for the alleviation of stress in oak [23]. As such the investigation of healthy oak roots for the isolation and identification of potential plant growth-promoting bacteria which could be used for biological stress release through rhizosphere action is becoming more frequently considered.

Using a polyphasic taxonomic approach, we performed a comprehensive classification of isolates collected from the rhizosphere soil surrounding the roots of healthy and symptomatic oak trees. The results gained in this study support the proposal of a novel species of *Leclercia* and a novel genus *Silvania* gen. nov. containing two novel species, *Silvania hatchlandensis* sp. nov. and *Silvania confinis* sp. nov.

## Results and discussion

### Genotypic identification

**Fig. 1:**
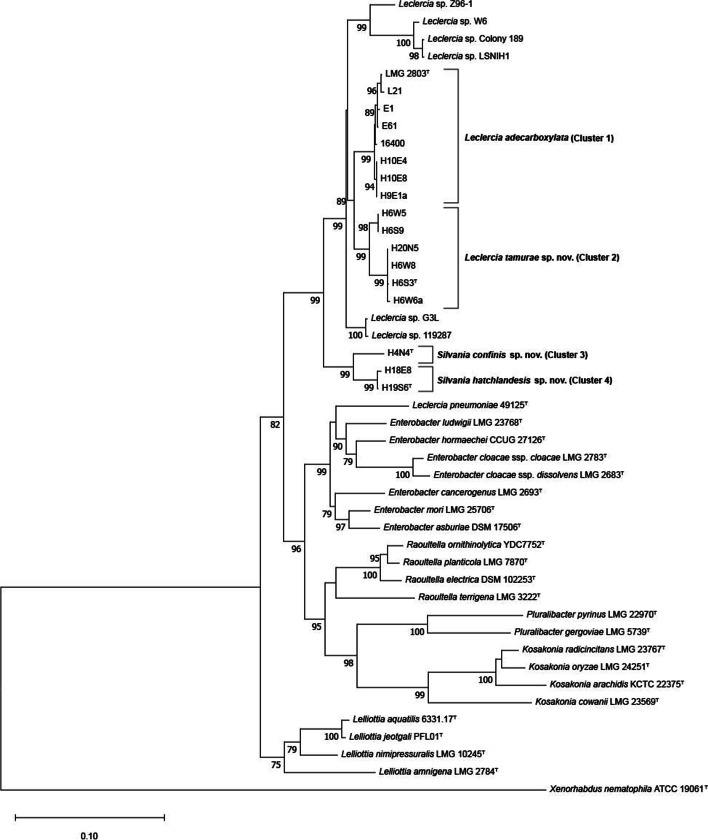
Maximum likelihood tree based on the concatenated partial gene sequences of *atpD*, *infB*, *gyrB* and *rpoB* from species of the proposed genus *Silvania* gen. nov., the novel species *Leclercia tamurae* sp. nov. and their closest phylogenetic neighbours. *Xenorhabdus nematophila* (ATTCC 190601^T^) was included as the outgroup. Percentages for bootstrap values (> 50%) following 1000 replicates are shown. The scale bar represents the number of substitutions per site. ^T^ denotes type strain

Bacterial strains were isolated from rhizosphere soil surrounding five healthy and two diseased native British oaks (*Q. petraea* and *Q. robur*) found at Hatchlands Park, Guildford, UK. The list of strains and isolation sources can be found in Table S1 (see Additional file 1). Multilocus sequencing analysis (MLSA) of the housekeeping genes *gyrB, rpoB, infB* and *atpD* for all 12 strains was performed to determine their taxonomic position*.* In the maximum likelihood phylogenetic tree based on the concatenated MLSA sequences (Fig. 1), the 12 strains were separated into four clusters. Cluster 1 contained three strains isolated from one healthy and one diseased tree, the type strain of *L. adecarboxylata* (LMG 2650^T^) and four strains assigned to *Leclercia* based on their whole genome sequences. Due to the lack of sequence variation (> 98.9% intra-species similarity for all four genes) and phylogenetic distance observed between the MLSA genes of these strains and the type strain of *L. adecarboxylata*, we concluded that they belong to this species. Cluster 2, situated proximal to the *L. adecarboxylata* cluster, contained strains isolated from three cardinal points around two healthy oaks, one in the parkland and another in the woodland, and was strongly supported by a bootstrap value of 99%, suggesting the strains belong to a novel *Leclercia* species. A higher degree of sequence variation was observed within Cluster 2 with strains exhibiting 96–100% sequence similarity across the four housekeeping genes, and the *gyrB* gene displaying the most heterogeneity. The *gyrB* sequence similarity between Cluster 2 strains and the type strain of *L. adecarboxylata* (LMG 2650^T^) was > 94.3%, and > 98.5% for the other three genes. Clusters 3 and 4 were contained in a clade with 99% bootstrap support and consisted of one and two strains, respectively, isolated from both healthy parkland oak and diseased woodland oak rhizosphere soil. This clade was situated on a separate lineage on the border of the *Leclercia* clade with a greater phylogenetic distance, suggesting the strains could belong to a potential novel genus with two novel species. An additional six strains, identified as *Leclercia* sp. in GenBank, clustered on three separate lineages in the *Leclercia* clade (G3L and 119,287; Z96–1 and W6; and Colony 189 and LSNIH1), suggesting they belong to several further potential novel *Leclercia* species. Of the six strains, Z96–1 has been incorrectly assigned to *L. adecarboxylata* [24], strain W6 was suggested as a novel species based on the computational analysis of its whole genome [25] and the remaining four have yet to be classified at the species level. Additionally, based on the MLSA phylogenetic tree, the taxonomic status of *Leclercia pneumoniae* 49125^T^ was unclear, as it clustered on the border of the *Enterobacter* clade, far removed from *Leclercia*.

Nearly complete 16S rRNA gene sequences (1344 bp) were obtained for the strains of the potential novel species (H6S3^T^, H6W5, H19S6^T^ and H4N4^T^). Strains H6S3^T^ and H6W5 (Cluster 2) showed 99.40–99.55% 16S rRNA gene pairwise sequence similarity to several *Enterobacter* species including the type strains of *E. huaxiensis, E. cancerogenus, E. sichuanensis and E. chengduensis* as well as 99.33% *to L. adecarboxylata*. The strains suggested as belonging to a potential novel genus by MLSA, H4N4^T^ (Cluster 3) and H19S6^T^ (Cluster 4), displayed highest pairwise similarity to *Lelliottia jeotgali* PFL01^T^ with 99.48 and 99.45% to *L. adecarboxylata* NBRC 102595^T^*,* respectively and a generally high similarity to *Lelliottia* and *Enterobacter* species. These results are not unusual for members of the order Enterobacterales which are known for being highly homogenous, meaning their taxonomic position at the species level cannot reliably be determined based on their 16S rRNA gene sequences [26]. This is reflected in the 16S rRNA gene maximum likelihood phylogenetic tree (Fig. S1, Additional file 2) where strains from the potential novel *Leclercia* species cluster within the *Enterobacter* clade, and species of the potential novel genus are situated on separate lineages in proximity to the *Lelliottia* clade.

To assess the genetic diversity between strains isolated from the oak rhizosphere, BOX and ERIC PCR were performed on all 12 strains. The results from the ERIC PCR allowed for greater discrimination between strains and demonstrated distinct patterns between *Leclercia* and the proposed novel genus (Fig. S2, Additional file 2). The fingerprints generated from *L. adecarboxylata* strains were easily differentiated from the novel Leclercia species, as were the patterns for the two species of the novel genus. Although identical clones were present within the novel *Leclercia* species genetic diversity was observed between these strains based on MLSA sequencing.

### Genomic characterisation

Whole genome sequencing was performed on five novel isolates from the four MLSA clusters (H10E4 – Cluster 1, H6S3^T^ and H6W5 – Cluster 2, H4N4^T^ – Cluster 3, and H19S6^T^ – Cluster 4). The genomes showed little variation, with the size and G + C DNA content ranging from 4.71–4.87 Mbp and 55.6–56.4 mol%, respectively. The genomes were submitted to GenBank under the BioProject numbers PRNJA837588 and PRNJA837589, and the genome features and accession numbers are listed in Table S2 (see Additional file 1). All sequenced genomes were found to be free of contamination following alignment and comparison of the 16S rRNA gene sequences obtained from both the whole genomes and Sanger sequencing.

**Fig. 2:**
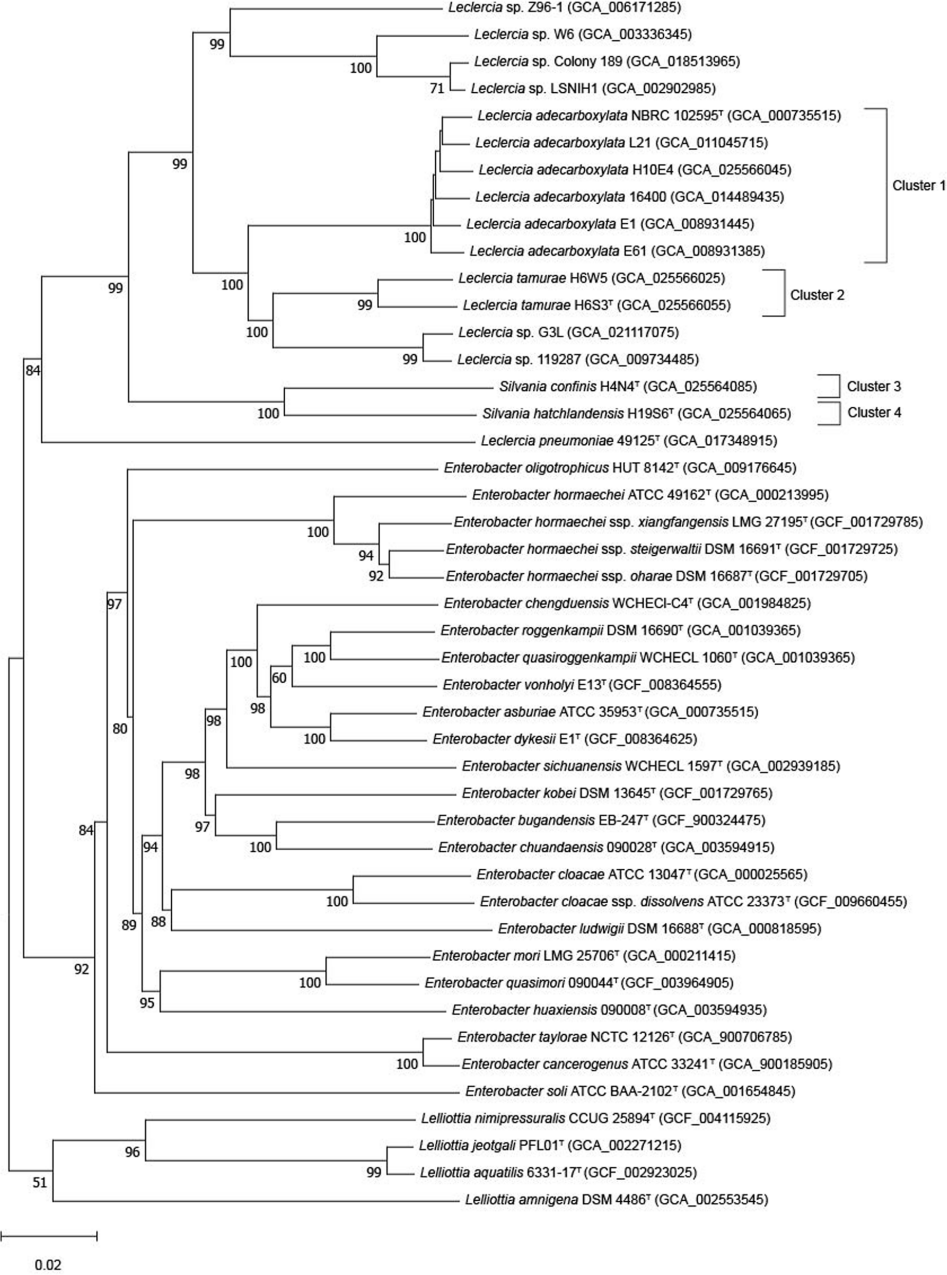
Phylogenomic tree of the proposed genus Silvania gen. nov., the novel species *Leclercia tamurae* sp. nov. and their closest phylogenetic neighbours. GBDP pseudo-bootstrap support values from 100 replicates (> 50%) are shown at the nodes, with the average branch support of 94.4%. Branch lengths are scaled from the d_5_ GBDP distance formula and the tree is rooted at the midpoint. ^T^ denotes type strain, and GenBank assembly numbers are shown in parentheses

The phylogenomic tree (Fig. 2), based on whole genome comparisons, supported the phylogeny demonstrated in the MLSA tree, with H10E4 confirmed as belonging to *L. adecarboxylata* along with other strains identified as *Lecleria* sp. in the MLSA tree. H6S3^T^ and H6W5 formed a well-supported cluster in the *Leclercia* clade, along with *Leclercia* strains GL3 and 119,287 from GenBank in a separate cluster which could constitute another novel species as observed in the MLSA tree. The other strains assigned to *Leclercia* in GenBank, Z96–1, W6, Colony 189 and LSNIH1, appear further removed from the main *Leclercia* clade, suggesting that they could constitute another novel genus, with three novel species. The two strains from Clusters 3 and 4 formed a clade with 100% bootstrap support, clearly distant from the *Leclercia* clade and did not contain any validly published type strain or reference strain confirming these strains constitute a novel genus. Finally, *Leclercia pneumoniae* 49125^T^ was furthest removed from the *Leclercia* clade on a separate lineage and did not cluster with any known type strain or reference strain.

To complement the phylogenomic comparison, a DNA similarity matrix was created through Average Nucleotide Identity (ANI), Average Amino Identity (AAI) and digital DNA-DNA hybridisation (dDDH) comparisons. The ANI and dDDH values are presented in Table 1, and the AAI values in Table S3 (See Additional file 1). H10E4 displayed dDDH values between 87.4–89.0%, ANI values between 98.4–98.6% and AAI values of 99% to the type strain of *L. adecarboxylata* LMG 2803^T^ and other strains identified as *L. adecarboxylata*, far exceeding the 70, 95 and 96% similarity values used to delimit species for dDDH, ANI and AAI [27–29]. Likewise, strains from Cluster 2 (H6S3^T^ and H6W5) demonstrated 89.9% dDDH, 98.6% ANI and 98% AAI values to each other but < 70% dDDH, < 95% ANI and 94–95% AAI values to *L. adecarboxylata*, confirming they belong to a single novel taxon. Finally, H4N4^T^ (Cluster 3) and H19S6^T^ (Cluster 4) were 45.6% similar based on dDDH, and 92.1 and 95% similar based on ANI and AAI, respectively. Both strains demonstrated lower values of < 35% dDDH, < 88% ANI and 90–91% AAI to strains of *Leclercia*, providing further support for their classification of a novel genus. Therefore, we propose *Leclercia tamurae* sp. nov. for strains in Cluster 2, and *Silvania* gen. nov. with *Silvania hatchlandensis* sp. nov. and *Silvania confinis* sp. nov. for strains in Clusters 3 and 4.

**Table 1:** Genome comparison values for digital DNA - DNA Hybridisation (dDDH – top right) and Average Nucleotide Identity (fastANI – bottom left)

dDDH fastANI	1	2	3	4	5	6	7	8	9	10	11	12	13	14	15	16	17
1	**100**	90.1	89.4	87.6	88.6	87.4	26.2	44.9	44.6	45.0	45.1	36.5	36.6	36.8	37.6	31.6	31.2
2	98.6	**100**	88.4	88.0	88.6	87.5	26.5	44.9	44.8	45.4	45.3	36.7	36.7	37.0	37.9	31.7	31.2
3	98.6	98.5	**100**	87.4	89.0	87.5	26.3	44.7	44.7	45.0	44.9	36.5	36.7	36.7	37.8	31.7	31.2
4	98.3	98.4	98.4	**100**	88.2	86.6	26.2	44.8	45.0	44.9	45.2	36.4	37.1	37.0	39.3	31.6	31.0
5	98.5	98.5	98.6	98.4	**100**	89.2	26.4	45.0	45.2	45.4	45.4	36.8	37.5	37.2	40.5	31.7	31.2
6	98.3	98.4	98.4	98.2	98.6	**100**	26.5	44.6	44.8	45.2	45.2	36.8	37.3	36.9	39.4	31.7	31.1
7	84.2	84.4	84.3	84.3	84.4	84.3	**100**	26.6	26.6	27.1	27.0	26.7	26.7	26.9	27.0	26.0	25.8
8	91.8	91.7	91.6	91.7	91.7	91.6	84.4	**100**	73.2	49.9	49.7	37.8	38.0	38.4	39.3	32.9	32.1
9	91.6	91.6	91.6	91.7	91.7	91.6	84.4	96.8	**100**	49.8	49.6	38.0	38.1	38.4	39.1	32.8	32.0
10	91.6	91.7	91.6	91.6	91.7	91.7	84.7	92.9	92.9	**100**	89.9	39.0	39.3	39.7	40.4	32.9	32.2
11	91.7	91.7	91.7	91.8	91.8	91.7	84.6	93.0	93.0	98.6	**100**	39.1	39.0	39.7	40.2	33.0	32.2
12	89.0	89.1	89.1	89.0	89.2	89.2	84.5	89.8	89.8	90.0	90.0	**100**	94.6	69.6	43.1	31.3	30.6
13	89.0	89.1	89.1	89.2	89.4	89.3	84.5	89.8	89.8	90.1	90.0	99.2	**100**	69.3	43.5	31.2	30.5
14	89.2	89.2	89.1	89.4	89.5	89.4	84.5	89.9	89.9	90.2	90.4	96.4	96.4	**100**	43.1	31.4	30.8
15	89.4	89.6	89.6	90.0	90.4	90.0	84.7	90.3	90.1	90.5	90.5	91.3	91.3	91.2	**100**	32.1	31.4
16	87.2	87.1	87.1	87.2	87.1	87.1	84.0	87.9	87.8	87.6	87.7	86.9	86.9	87.0	87.5	**100**	46.5
17	86.7	86.8	86.8	86.8	86.8	86.6	83.8	87.4	87.3	87.3	87.3	86.6	86.5	86.6	87.0	92.1	**100**

Strains which exceed the cut of values used for species delimitation are shown in shaded boxes (> 70% dDDH or > 95% ANI). 1 = *Leclercia adecarboxylata* NBRC 102595^T^ (GCA_001515505), 2 = *Leclercia adecarboxylata* L21 (GCA_011045715), 3 = *Leclercia adecarboxylata* H10E4 (GCA_025566045), 4 = *Leclercia adecarboxylata* 16,400 (GCA_014489435), 5 = *Leclercia adecarboxylata* E1 (GCA_008931445), 6 = *Leclercia adecarboxylata* E61 (GCA_008931385), 7 = *Leclercia pneumoniae* 49125^T^ (GCA_018987305), 8 = *Leclercia tamurae* H6S3^T^ (GCA_025566055), 9 = *Leclercia tamurae* H6W5 (GCA_025566025), 10 = *Leclercia* sp. G3L (GCA_021117075), 11 = *Leclercia* sp. 119,287 (GCA_009734485), 12 = *Leclercia* Colony 189 (GCA_018513965), 13 = *Leclercia* sp. LSNIH1 (GCA_002902985), 14 = *Leclercia* sp. W6 (GCA_003336345), 15 = *Leclercia* sp. Z96–1 (GCA_006171285), 16 = *Silvania hatchlandensis* H19S6^T^ (GCA_025564065), 17 = *Silvania confinis* H4N4^T^ (GCA_025564085)

The dDDH, ANI and AAI values for the additional *Leclercia* strains support the phylogenies of the MLSA and phylogenomic trees. Strains GL3 and 119,287 demonstrated similarity values indicating they belong to a novel species closely related to *L. adecarboxylata* and *L. tamurae* sp. nov. Species of *Leclercia* exhibited 94–95% AAI similarity, while the two novel species of *Silvania* gen. Nov. were 95% similar based on AAI. In contrast, strains Z96–1, W6, Colony 189, LSNIH1 and *L. pneumoniae* 49125^T^ were less related to species of *Leclercia* with AAI values ranging from 88 to 94%), suggesting these strains most likely belong novel genera, although further work would be required to fully understand their taxonomic position. It is worth noting that *L. pneumoniae* 49125^T^ was least related to all strains of *Leclercia* species displaying AAI values of 88–89%. There is no currently accepted AAI cut-off for delineating genera, although several values have been suggested [30–32]. However, these do not appear to be stringent enough for members of the *Enterobacteriaceae* and a comprehensive study of the family is needed before a genus delineation cut-off can be proposed.

### Genomic features

**Fig. 3:**
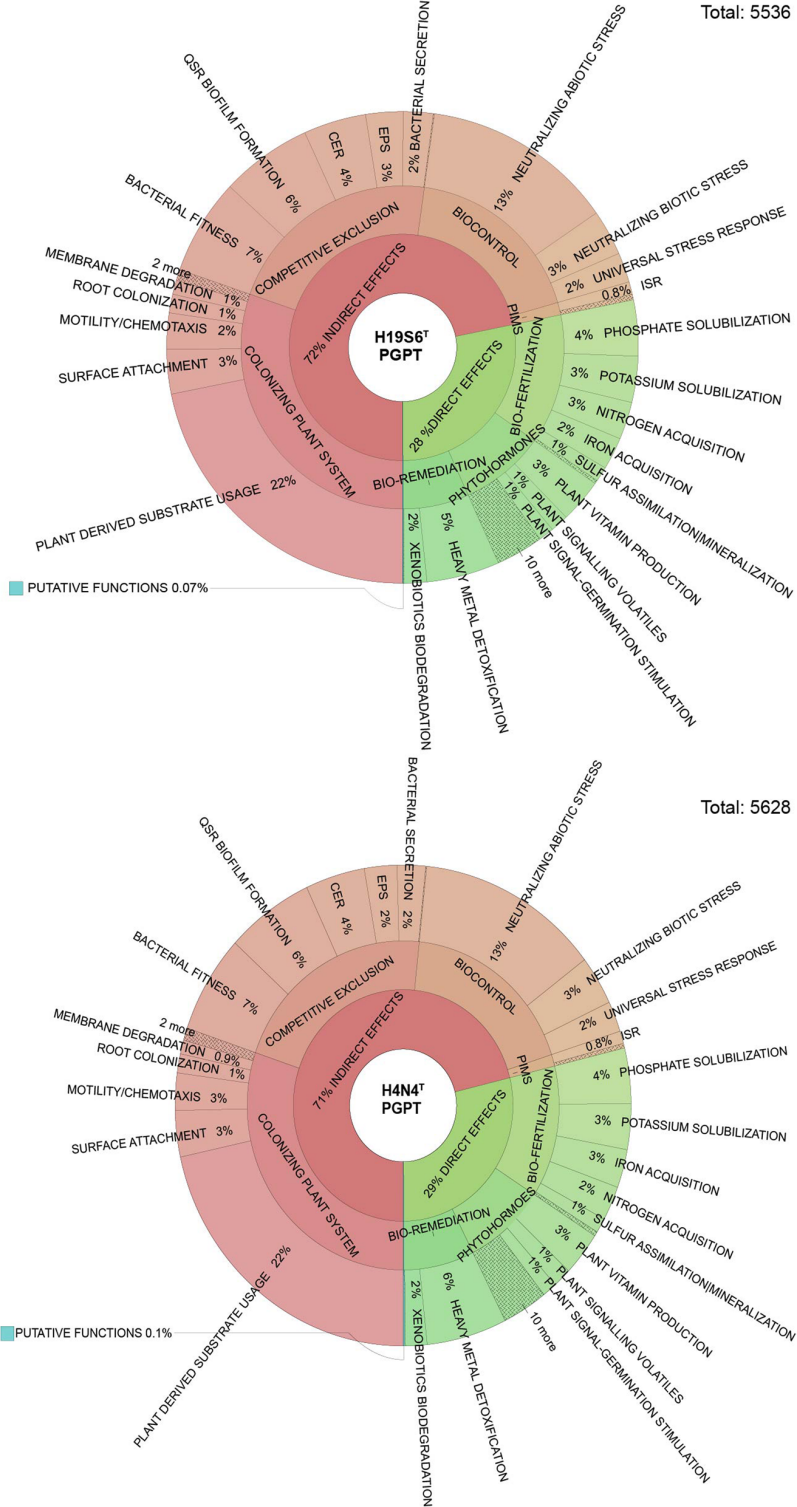
Krona plot representation of the major plant growth-promoting traits found in *Silvania hatchlandensis* sp. nov. (H19S6^T^) and *Silvania confinis* sp. nov. (H4N4^T^). Identification of PGPTs was performed by BlastP and HMMER annotation against the PGPT-BASE. Text files of the annotation were downloaded, and Krona plots were made using the ‘ktImportText’ command in Bioconda. Depth of annotation is shown to level three of six, excluding pathways, gene names and accession numbers. QSR = Quorum sensing response, CER = Cell envelope remodelling, EPS = Exopolysaccharide production, PIMS = Plant immune system stimulation and ISR = Induction of systemic resistance

**Fig. 4:**
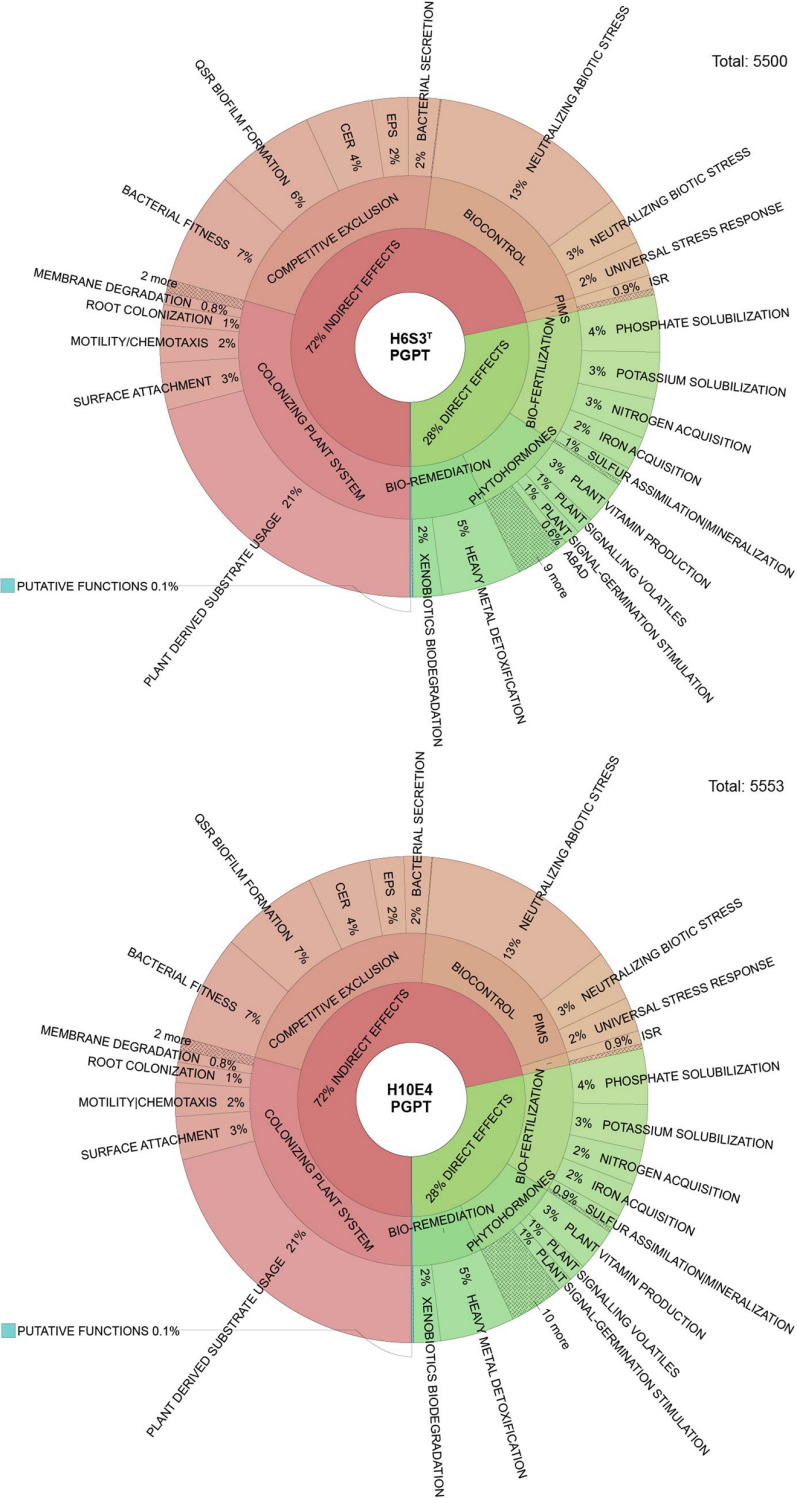
Krona plot representation of the major plant growth-promoting traits found in *Leclercia adecarboxylata* (H10E4) and *Leclercia tamurae* sp. nov. (H6S3^T^). Identification of PGPTs was performed by BlastP and HMMER annotation against the PGPT-BASE. Text files of the annotation were downloaded, and Krona plots were made using the ‘ktImportText’ command in Bioconda. Depth of annotation is shown to level three of six, excluding pathways, gene names and accession numbers. QSR = Quorum sensing response, CER = Cell envelope remodelling, EPS = Exopolysaccharide production, PIMS = Plant immune system stimulation, ISR = Induction of systemic resistance and ABAD = Abscisic acid degradation

To investigate the potential of *L. adecarboxylata*, *L. tamurae* sp. nov. and species of *Silvania* gen. nov. as plant growth-promoting bacteria (PGPB) playing a positive role in the soil, their plant growth-promoting traits (PGPT) were investigated computationally. The results from the DIAMOND MEGAN pipeline comparison against the PLant-associated BActeria web resource (PLaBAse) database revealed larger numbers of important plant interaction proteins through the PGPT viewer and KEGG orthology viewer. The resulting PGPT data showed that each submitted annotated genome had between 5500 and 5638 PGPTs aligned to known proteins. The majority produced indirect effects such as stress relief and biocontrol, competitive exclusion and genes involved in colonising the plant system. Of the direct effects, the main categories of the genes were involved in bioremediation, phytohormone production and biofertilisation. Figures 3 and 4 show the Krona plots for the type strains of the novel species and *L. adecarboxylata* H10E4. Traits of interest included potassium and phosphate solubilisation, nitrogen and iron acquisition, sulphur assimilation and carbon dioxide fixation, features which all directly aid plant growth by increasing nutrient availability. 13% of the PGPT involved abiotic stress responses to neutralise salinity, osmotic, nitrosative/oxidative, herbicidal, and acidic stress, which are predisposing environmental factors in decline disease [20]. It has been demonstrated previously that highly acidic soils are known to contribute to AOD symptoms [33], especially in parkland systems where many of the strains in the present study were isolated from. A small number of zinc heavy metal resistance genes responsible for *L. adecarboxylata* MO1s plant growth-promoting association [34] were identified in all species, although most of the heavy metal resistance genes were related to iron. Few differences could be seen between the *Leclercia* and *Silvania* gen. nov. species although H4N4^T^ had more alignments and the largest number of PGPTs identified. However, given their highly conserved AAI values of 90–91%, this is unsurprising and a further implication of their phylogenetic relatedness. The conclusive statement for each strain annotated genome comparison against plant bacterial only interaction factors (proteins) or PIFAR, suggested that the novel species were all capable of interaction with plants, but the identified interaction factors were related to virulence. 31–32% of factors were toxins (syringomycin and toxoflavin), 17–19% were exopolysaccharides (namely amylovoran), 8–9% of *Silvania* gen. nov. and 11–12% of *Leclercia* factors were for detoxification (of plant compounds such as isothiocyanate), and ~ 15% were adhesion and metabolism genes. Between 0.6–0.9% (*Leclercia*) and 2% (*Silvania* gen. nov.) of the identified bacterial plant interaction markers were plant cell wall degrading enzymes which are key markers of phytopathogens. The features identified through PIFAR such as EPS, toxins and PCWDE implicate the novel isolates as having pathogenic potential. These genes are associated with the invasion, colonisation and degradation of plant tissue [35]. However, many of these genes are also used by PGPB for the colonisation of plants, where they continue to have a positive effect. Nonetheless, the identified pathogenicity traits complicate the potential role of these isolates as PGPB concerning oak [36].

Results from the comparison to the virulence factor database (VFDB), however showed 126–140 proteins from the novel strains were aligned to known virulence proteins from other pathogens with the vast majority related to motility, immunomodulation and adhesion. Some Type VI Secretion System (T6SS) effector delivery system proteins were identified, although no complete set of the assembly proteins and no secreted effector proteins were identified in the alignments. These results imply that the novel isolates have low pathogenic potential, although experimental pathogenicity trials with the type strains should be performed for conclusive understanding of their pathogenic potential.

Overall, we can speculate on the isolates’ potentially positive role in the rhizosphere through several important direct PGPT genes such as heavy metal detoxification, biofertilisation and phytochemical signalling which all aid plant growth and resilience. Alongside the direct effects are other indirectly positive PGPTs such as stress relief for osmotic, heat, salinity and competitive exclusion genes. However, based on the alignments made in both the VFDB and through the PIFAR database the novel isolates here all contain genes related to virulence such as motility, adhesion, and Immune modulation genes. These genes could implicate a potential for pathogenicity, although all genes identified could also be utilised by PGPB for colonisation of the plant endosphere. While we cannot conclude on the role these isolates play in this niche, it is probable based on comparison to *L. adecarboxylata* MO1 that the novel strains isolated in the present study promote plant growth through their action in the rhizosphere, especially in relation to heavy metal detoxification [34].

### Physiology and chemotaxonomy

#### Morphology of cells and colonies

All strains identified as *Leclercia* were straight rods averaging 1.38 × 2.26 μm, while *Silvania* gen. nov. strains were short straight rods averaging 1.31 × 1.81 μm. Cells are motile with peritrichous flagella and appear singly or in pairs (Fig. S3, Additional file 2). On tryptone soy agar (TSA) all strains tested appeared as circular, cream-coloured, convex colonies between 2 and 4 mm in diameter with entire, slightly undulate margins. All strains were observed changing from cream to yellow pigmented which is a known feature associated with *Leclercia*, although the time and conditions required for the pigment to form were not consistent [37].

#### Growth characteristics

*Leclercia* species grew from 10 to 41 °C, while species of *Silvania* gen. nov. grew at 4–37 °C but not at 41 °C. The pH range at which growth was observed showed no difference between strains from both genera, with consistent growth seen from pH 6–9. All strains from both genera grew in a supplemented salt range of 1–7%, with the exception of *L. adecarboxylata* LMG 2803^T^, *L. tamurae* sp. nov. H6W6a and H6W8, and *S. confinis* sp. nov. H4N4^T^ which could not grow at 7%. All strains were recorded as negative for oxidase and positive for catalase production, which are key descriptive factors of the family *Enterobacteriaceae*.

#### Antibiotic testing

Antibiotic resistance for all strains from both genera was recorded for penicillin V and G, while susceptibility was recorded for tetracycline, ampicillin, chloramphenicol, colistin sulphate, streptomycin, cefotaxime, ciprofloxacin, cefepime, gentamycin and kanamycin.

#### Substrate utilisation and enzyme activity

The new species and genus described in this paper all present phenotypically unique traits tested with commercial kits, which can be used for their differentiation from each another and their closest relatives. *Leclercia* and *Silvania* gen. nov. can be distinguished based on a number of traits including fermentation of D-arabinose and utilisation of p-hydroxy-phenylacetic acid and fusidic acid. *L. tamurae* sp. nov. can be differentiated from *L. adecarboxylata* based on the positive reaction to sorbitol and the inability to utilise D-adonitol or D-arabitol among other traits, while *Silvania* gen. nov. species can be discriminated by reactions to indole production, rhamnose and sucrose fermentation and pectin utilisation. Tables 2 and 3 show the most useful phenotypic characteristics used for the differentiation between species of *Leclercia* and *Silvania* gen. nov., respectively and Table 4 shows those for the differentiation between the two genera. Positive phenotypic characteristics shared by all current members of *Leclercia* and *Silvania* gen. nov. are listed in Table S4.

**Table 2:** Key phenotypic characteristics for differentiation of *Leclercia* species and *Leclercia tamurae* sp. nov.

Reaction	*Leclercia adecarboxylata* (*n* = 4)	*Leclercia pneumoniae* 49125^T^	*Leclercia tamurae* (*n* = 5)
sorbitol	–	–	+
sucrose	V^a^	–	–
Acidification of:			
D-adonitol	+	ND	–
dulcitol	V^a^	ND	+
methyl-α-D-glucopyranoside	–	ND	V^b^
D-trehalose	+	+	V^a^
D-raffinose	V^a^	ND	–
D-lyxose	V^b^	ND	V^a^
D-arabitol	+	–	–
potassium 2-ketogluconate	+	ND	–
potassium 5-ketogluconate	–	–	V^b^
palatinose	V^a^	–	–
malonate	V^b^	+	+
*N*-acetyl-β-glucosaminidase	–	–	V^a^
Utilisation of:			
sucrose	–	–	V^a^
stachyose	V^a^		–
D-salicin	+	ND	V^a^
3-methyl glucose	–	ND	V^a^
D-aspartic acid	+	ND	–
pectin	V^a^	ND	–
citric acid	–	ND	V^a^
α-keto-glutaric acid	–	ND	–
D-malic acid	V^a^	ND	V^b^
potassium tellurite	–	ND	V^b^
tween 40	+	ND	V^a^
α-hydroxy-butyric acid	V^a^	ND	V^a^
β-hydroxy-D,L-butyric acid	–	ND	V^b^
formic acid	+	ND	V^b^
Resistant to:			
D-serine	–	ND	V^a^
nalidixic acid	V^b^	ND	+
troleandomycin	–	ND	V^a^

+, positive reaction; −, negative reaction; V, variable within species; ^a^, positive for type strain; ^b^, negative for type strain

**Table 3:** Key phenotypic characteristics for differentiation of *Silvania hatchlandensis* sp. nov. and *Silvania confinis* sp. nov.

Reaction	*Silvania hatchlandensis* (*n* = 2)	*Silvania confinis* H4N4^T^
indole production	+	–
rhamnose	+	–
sucrose	+	–
Acidification of:		
methyl-α-D-mannopyranoside	V^b^	–
methyl-α-D-glucopyranoside	V^b^	–
D-lyxose	–	+
D-tagatose	–	+
phenol red	+	–
β-glucuronidase	+	–
malonate	V^b^	–
Utilisation of:		
stachyose	+	–
*N*-acetyl-D-galactosamine	+	–
L-pyroglutamic acid	+	–
pectin	+	–
quinic acid	+	–
α-keto-glutaric acid	+	–
D-malic acid	+	–
Resistant to:		
D-serine	–	+
troleandomycin	–	+
nalidixic acid	–	+
potassium tellurite	–	+

+, positive reaction; −, negative reaction; V, variable within species; ^a^, positive for type strain; ^b^, negative for type strain

**Table 4:** Key phenotypic characteristics for differentiation between *Leclercia* and *Silvania* gen. nov.

Reaction	Leclercia* (*n* = 9)	*Silvania* (*n* = 3)
indole production	+	V^a^
sorbitol	V^a^	+
rhamnose	+	V^a^
sucrose	V^b^	V^a^
Acidification of:		
D-arabinose	–	+
D-adonitol	V^b^	–
methyl-α-D-mannopyranoside	–	V^b^
methyl-α-D-glucopyranoside	V^b^	V^b^
D-raffinose	V^b^	+
D-lyxose	V^a^	V^b^
D-tagatose	–	V^b^
D-arabitol	V^b^	–
potassium 2-ketogluconate	V^b^	–
potassium 5-ketogluconate	V^b^	+
phenol red	+	V^a^
palatinose	V^b^	–
β-glucuronidase	–	V^a^
malonate	V^a^	V^b^
*N*-acetyl-β-glucosaminidase	V^a^	–
Utilisation of:		
sucrose	V^a^	+
stachyose	V^b^	V^a^
*N*-acetyl-D-galactosamine	–	V^a^
3-methyl glucose	V^a^	+
D-aspartic acid	V^b^	+
D-serine	V^a^	+
L-pyroglutamic acid	V^a^	V^a^
pectin	V^b^	V^a^
quinic acid	–	V^a^
p-hydroxy-phenylacetic acid	+	–
citric acid	V^a^	+
α-keto-glutaric acid	–	V^a^
D-malic acid	V^a^	V^a^
α-hydroxy-butyric acid	V^a^	–
β-hydroxy-D,L-butyric acid	V^b^	–
formic acid	V^b^	–
Resistant to:		
fusidic acid	+	–
D-serine	+	V^b^
troleandomycin	V^a^	V^b^
nalidixic acid	V^a^	V^b^
potassium tellurite	V^b^	V^b^

+, positive reaction; −, negative reaction; V, variable within species; ^a^, positive for type strain; ^b^, negative for type strain. * *Leclercia adecarboxylata* and *Leclercia tamurae* sp. nov.

#### Fatty acid methyl ester profiles

Based on the results generated by the Sherlock Microbial Identification System Version 6.4 (MIDI Inc.), the major fatty acids (above 10% relative abundance) were C_16:0_, C_18:1_ ω7*c,* and summed feature 3 (C_16:1_ ω7c and/or C_16:1_ ω6c). The fatty acid profiles for each strain can be seen in Table 5. Minor differences can be observed between amounts of C_18:1_ ω7*c* which is higher in *Leclercia* species and summed feature 3 (C_16:1_ ω7c and/or C_16:1_ ω6c) which is higher in *Silvania* gen. nov. species.

**Table 5:** The major fatty acid methyl ester (FAME) average % peaks and standard deviation for *Leclercia* and *Silvania* gen.nov.

	*Leclercia adecarboxylata* (*n* = 2)	*Leclercia tamurae* (*n* = 4)	*Silvania hatchlandensis* H19S6^T^	*Silvania confinis* H4N4^T^
Saturated fatty acids				
C_12:0_	3.7 (± 0.0)	3.8 (± 0.2)	3.5	2.9
C_14:0_	5.3 (± 0.1)	5.4 (± 0.1)	5.5	5.2
C_16:0_	26.4 (±1.1)	28.4 (± 1.1)	25.8	24.4
Cyclopropane fatty acids				
C_17:0_ cyclo	7.1 (± 1.9)	9.3 (± 1.9)	6.7	2.8
Unsaturated fatty acids				
C_18:1_ ω7*c*	21.4 (± 0.2)	20.0 (± 0.2)	18.4	14.9
Summed features				
2: C_14:0_ 3-OH and/or iso-C_16:1_	7.5 (± 0.0)	8.7 (± 0.0)	7.4	7.8
3: C_16:1_ ω7*c* and/or C_16:1_ ω6*c*	24.3 (± 1.2)	22.0 (± 1.2)	27.2	35.7

## Conclusion

Even though *Leclercia* was a monospecific member of the *Enterobacteriaceae* since being defined in the 1960s until 2022, it has remained an interesting genus with clinical and environmental importance including plant-growth promoting abilities when added to the rhizosphere. The work presented here provides evidence that both the type species of the genus and a number of phylogenetically related species are associated with the rhizosphere of both healthy and diseased oaks suffering from AOD.

The genomic, genotypic, chemotaxonomic and phenotypic data suggests the strains investigated in this study represent three novel species, two of which belong to a novel genus. As such the following descriptions are proposed; *Leclercia tamurae* sp. nov. (type strain = H6S3^T^ = LMG 32617^T^ = CCUG 76176^T^), *Silvania* gen. nov. with the type species as *Silvania hatchlandensis* sp. nov (type strain = H19S6^T^ = LMG 32608 ^T^ = CCUG 76185 ^T^) and *Silvania confinis* sp. nov (type strain = H4N4^T^ = LMG 32607 ^T^ = CCUG 76175 ^T^) and the amendment of the genus and type species descriptions for *Leclercia*. The addition of a new species to the genus *Leclercia* furthers our understanding of this clinically and environmentally important genus. Moreover, the taxonomic position of *Leclercia* has always been distantly removed from other genera of enteric bacteria within phylogenomic and phylogenetic trees. Through the addition of the closely related genus *Silvania* gen. nov. composed of two species, the wider taxonomic relationship of both genera within the family *Enterobacteriaceae* can be further understood.

### Emended description of the genus *Leclercia*

*Leclercia* (Le.clerc’ i.a. M.L. fem. n. *Leclercia* was named to honour H. Leclerc, a French bacteriologist, who first described and named this organism *Escherichia adecarboxylata* in 1962, and who made many other contributions to enteric bacteriology).

Gram-negative rods, ranging from 1.39–1.54 μm wide and 2.01–3.06 μm long. All strains possess fimbriae and are motile by peritrichous flagella, and are oxidase negative, catalase positive, facultative anaerobes. After 48 h on TSA, all species appear as cream-coloured, circular, convex colonies between 2 and 3 mm in diameter with entire, slightly undulate margins. After longer periods of incubation some strains may develop a yellow diffusible pigment, although the conditions required are not consistent. Growth is observed from 10 to 41 °C for all strains, although some strains can grow at 4 °C, with optimal growth observed between 30 and 35 °C. The majority of strains grow at pH 6–9 and at supplemented salt concentrations of 1–8%, with some strains only able to grow up to 7%. Positive for β-galactosidase and indole production. Negative for arginine dihydrolase, lysine decarboxylase, ornithine decarboxylase, citrate utilization, H_2_S production, urease, tryptophan deaminase, acetoin production (VP) and gelatinase. Nitrite is reduced to nitrate. Production of β-glucosidase and α-galactosidase, acidification of galacturonate and phenol red, (ID 32). Resistant to 1% sodium lactate, fusidic acid, D-serine, rifamycin, lincomycin, guanidine HCl, niaproof 4, vancomycin, tetrazolium violet, tetrazolium blue, lithium chloride, aztreonam and sodium butyrate (Biolog Gen III).

The major fatty acids are C_16:0_, C_18:1_ ω7*c* and summed feature 3 (C_16:1_ ω7c and/or C_16:1_ ω6c). The DNA G + C content ranges from 55.8–56.4 mol%.

The type species is *Leclercia adecarboxylata*.

### Emended description of *Leclercia adecarboxylata*

The description is as given above for the genus with the following additional characteristics.

In addition to the carbon sources listed in Table S4, acid is produced from D-adonitol, D-arabitol and potassium 2-ketogluconate; and D-salicin, D-aspartic acid and tween 40 are utilised. Variable for the fermentation of saccharose, dulcitol, D-raffinose and D-lyxose; the acidification of palatinose and the production of malonate. Utilisation of the following carbon sources is variable: stachyose, L-pyroglutamic acid, pectin, D-malic acid and α-hydroxy-butyric acid. Variable resistance to nalidixic acid is observed.

The DNA G + C content of the type strain is 55.8 mol%.

The type strain is *Leclercia adecarboxylata* (ATCC 23216; CIP 82.92; DSM 30081; DSM 5077; HAMBI 1696; JCM 1667; LMG 2803; NBRC 102595; NCTC 13032).

### Description of *Leclercia tamurae* sp. nov.

*Leclercia tamurae* (ta.mu’rae. N.L. gen. Masc. n. *tamurae*, of Tamura, named in honour of Kazumichi Tamura for his role in defining the genus *Leclercia*).

The description is as given above for the genus with the following additional characteristics.

After 48 h on TSA, colonies are circular, matte, brittle and cream-coloured with slightly undulate margins with an average diameter of 3 mm. All strains are capable of forming the yellow pigmentation associated with *Leclercia*, although not within a set timeframe.

In addition to the carbon sources listed in Table S4, acid is produced from sorbitol and dulcitol and acidification of malonate is observed. Variable features include the fermentation of methyl-α-D-glucopyranoside, D-trehalose, D-lyxose and potassium 5-ketogluconate, and the production of *N*-acetyl-β-glucosaminidase. Utilisation of the following carbon sources is variable: sucrose, D-salicin, 3-methyl glucose, D-serine, L-pyroglutamic acid, citric acid, D-malic acid, tween 40, α-hydroxy-butyric acid, β-hydroxy-D, L-butyric acid and formic acid. Variable resistance to troleandomycin and potassium tellurite is observed.

The DNA G + C content of the type strain is 56.4 mol%.

The type strain is H6S3^T^ (= LMG 32609^T^ = CCUG 76176^T^) and was isolated from healthy *Quercus robur* rhizosphere soil in Hatchlands, Guildford, UK.

### Description of *Silvania* gen. nov

*Silvania* (Sil.va’ni.a. N.L. fem. n. *Silvania*, named after Silvanus the Roman deity of woodlands).

Gram-negative, straight rods (1.2–1.4 × 1.6–2.0 μm) and motile by peritrichous flagella. Cells appear singly or in pairs. Oxidase negative, catalase positive facultative anaerobes. Colonies appear as cream-coloured, convex circles with raised entire margins and a diameter of 3–4 mm on TSA. Growth is observed between 4 and 37 °C with an optimum growth temperature of 30 °C. Positive for β-galactosidase, negative for arginine dihydrolase, lysine decarboxylase, ornithine decarboxylase, citrate utilization, H_2_S production, urease, tryptophan deaminase, acetoin production and gelatinase. Nitrite is reduced to nitrate. Positive for the acidification of galacturonate and production of β-glucosidase and α-galactosidase (ID 32). Resistance to 1% sodium lactate, rifamycin, lincomycin, guanidine HCl, niaproof 4, vancomycin, tetrazolium violet, tetrazolium blue, lithium chloride, aztreonam and sodium butyrate is observed.

Variable features of the genus include indole production; fermentation of rhamnose, saccharose, methyl-α-D-mannopyranoside, methyl-α-D-glucopyranoside, D-lyxose, D-tagatose; acidification of phenol red and production of β-glucuronidase and malonate. Utilisation of the following carbon sources is variable: stachyose, *N*-acetyl-D-galactosamine, fusidic acid, D-serine, L-pyroglutamic acid, pectin, quinic acid, α-keto-glutaric acid and D-malic acid. Variable resistance to troleandomycin, nalidixic acid and potassium tellurite is observed. The major fatty acids are C_16:0_, C_18:1_ ω7*c *and summed feature 3 (C_16:1_ ω7c and/or C_16:1_ ω6c).

The DNA G + C content ranges from 55.7 - 55.9 mol%.

The type species is *Silvania hatchlandensis.*

### Description of *Silvania hatchlandensis*

*Silvania hatchlandensis* (hatch.lan.den’sis. N.L. fem. Adj. *hatchlandensis*, pertaining to Hatchlands the national park in Guildford, UK where the strains were isolated from).

The description is as given above for the genus with the following additional characteristics.

Cells are on average 1.25 × 1.94 μm in size. After 48 h on TSA, the colonies appear as slightly raised circles with raised entire margins and an average diameter of 4 mm. Positive for indole production (API 20 and API 50 CHB/E), the acidification of phenol red and the production of β-glucuronidase (ID 32). Variable features of the species include the fermentation of methyl-α-D-mannopyranoside and methyl-α-D-glucopyranoside; the production of malonate. In addition to the carbon sources listed in Table S4, *N*-acetyl-D-galactosamine, L-pyroglutamic acid, quinic acid, α-keto-glutaric acid and D-malic acid are utilised.

The DNA G + C content of the type strain is 55.9 mol%.

The type strain is H19S6^T^ (= LMG 32608^T^ = CCUG 76185^T^) and was isolated from diseased *Quercus robur* rhizosphere soil in Hatchlands, Guildford, UK.

### Description of *Silvania confinis*

*Silvania confinis* (con.fi’nis. L. fem. Adj. *confinis*, adjoining/akin, referring to the close phylogenetic relationship to the type species of the genus).

The description is as given above for the genus with the following additional characteristics.

Cells are on average 1.37 × 1.68 μm in size. After 48 h on TSA, the colonies appear as slightly raised circles with raised entire margins and an average diameter of 3 mm. In addition to the carbon sources listed in Table S4, acid is produced from D-lyxose and D-tagatose. Resistance to D-serine, troleandomycin, nalidixic acid and potassium tellurite is observed.

The DNA G + C content of the type strain is 55.7 mol%.

The type strain is H4N4^T^ (= LMG 32607^T^ = CCUG 76175^T^) and was isolated from healthy *Quercus robur* rhizosphere soil in Hatchlands, Guildford, UK.

## Methods

### Isolation and DNA extraction

Samples were collected from Hatchlands Park, Guildford, UK from asymptomatic (healthy) and AOD symptomatic oaks (both *Q. robur* and *Q. petraea*) from both the parkland and woodland. Twenty oak trees were selected in a paired fashion (minimal spatial separation between healthy and diseased pairs in parkland and woodland). Rhizosphere soil samples were collected from the cardinal points around each of the 20 trees comprising a total of 80 samples. Following collection, soil was placed in sterile sample bags and transported directly to the University of the West of England, where they were stored at − 20 °C until processed.

Rhizosphere soil was removed from the root by hand and passed through a 2 cm sieve to remove any debris. DNA was extracted from the roots using the Extract ‘n Amp™ Plant PCR kit (XNAP2; Sigma) in which 1.5 cm of fine root (≤ 2 mm) were ground, incubated in 100 μL of extraction buffer at 95 °C for 10 minutes and then diluted in 100 μL of dilution buffer. Extracted root DNA was used in the amplification of the actin gene in a Loop-Mediated Isothermal Amplification reaction (LAMP) to confirm the identity of root samples as originating from oak (Bridget Crampton, personal communication). An isolation strategy originally designed for the recovery of enteric bacteria from food was utilised for the isolation of rhizosphere soil bacteria [38]. 10 g of rhizosphere soil from confirmed oak roots was suspended in 100 mL of *Enterobacteriaceae* enrichment broth (EE broth, Thermo Scientific), disrupted at 1150 RPM by a magnetic stirring rod for 10 minutes and the resulting suspension was placed in a shaking incubator at 250 RPM at 28 °C for 48 hours. Suspensions were removed from the incubator, allowing the sediment to settle before being diluted four-fold in ¼ strength Ringers (Oxoid). 100 ml of dilution was spread-plated on Eosin Methyl Blue agar (EMB, Merck) and incubated at 28 °C for 48 hours, both aerobically and anaerobically for the isolation of single colonies. All strains were stored in 50% glycerol at − 80 °C and subsequently cultured on Luria-Bertani (LB, Oxoid) agar and nutrient agar (NA, Oxoid) or in LB and nutrient broth incubated at 28 °C. Table S1 (see Additional file 1) lists the strains isolated and investigated in this study.

Alkalic lysis [39] was used to isolate genomic DNA by boiling bacterial cells isolated from a single colony in 0.05 mol l^− 1^ NaOH / 0.25% SDS for 15 min, followed by 10-fold dilution of the lysate and centrifugation to pellet cell debris. The isolated genomic DNA was used in subsequent PCR reactions, and was stored at − 20 °C.

### Genotypic characterisation

PCR amplification and sequencing was performed on housekeeping genes, *gyrB, rpoB, infB* and *atpD*, as described by Brady et al [40] and the 16S rRNA gene using the conditions and primers from Coenye et al. [41]. However, alternative sequencing primers with increased degeneracy were used for inf*B* and atp*D*, see Additional file 1, Table S5.

To ensure coverage in both directions for the 16S rRNA and housekeeping genes sequenced for isolates, consensus sequences were generated in UGENE V 38.1 [42]. Sequences for the closest phylogenetically related species, as well as for strains already assigned to the genus *Leclercia*, were downloaded from GenBank via BLAST [43] and added to the dataset. Sequences were aligned via Clustal-W and trimmed in MEGA X v11.0 [44] to the following lengths: *gyrB* – 742 bp, *rpoB* – 637 bp, *infB* – 615 bp, *atpD* – 642 bp and 16S rRNA gene – 1344 bp. Sequences for the housekeeping genes were conceptually translated in MEGA to ensure they were in the correct reading frame and that no errors were made from alignment gaps. 16S rRNA gene pairwise similarity for the potential novel species was calculated using the EZBioCloud server [45]. Finally, smart model selection [46] was applied to the concatenated housekeeping gene and 16S rRNA datasets using the online PhyML server [47]. Maximum likelihood phylogenetic analysis was performed on both the MLSA and 16S rRNA gene datasets in MEGA X with 1000 bootstrap replicates to assess the reliability of the clusters generated.

ERIC and BOX PCR using the primers and protocol from Versalovic, Koeuth and Lupski, [48] were used to assess the genetic diversity between strains. The resulting amplicons were separated for ~ 3 h in 1.5% agarose at 50 V (2 V/cm).

### Genomic characterisation

DNA was extracted from (H6S3^T^, H6W5, H10E4, H19S6^T^ and H4N4^T^) by enzymatic cell lysis with lysozyme and RNase A, purified on Solid Phase Reversible Immobilisation beads, followed by sequencing on the Illumina HiSeq platform by Microbes-NG (Birmingham, UK). Trimmomatic 3.0 was used to trim adapters at a sliding window quality cut-off of Q15 [49] and SPAdes 3.11.1 was used for the de novo assembly of contigs [50].

Pairwise comparisons of the genomes were calculated using Genome Blast Distance Phylogeny (GBDP) with the Type Strain Genome Server [51] and the ‘trimming’ algorithm with the distance formula *d*_*5*_ and 100 bootstrap replicates [52]. The resulting intergenomic distances were used to draw a genome caption tree using FastME 2.1.6.1 with the branch lengths scaled using the formula *d*_*5*_ [53]_._ Subtree Pruning and Regrafting (SPR) were used to ensure the best topology for the final tree, which was rooted at the midpoint [54].

Average Nucleotide Identity (ANI) were calculated in FastANI [55], Average Amino Identity (AAI) was calculated through the Kostas lab Genome distance calculator [56] and dDDH results were obtained using the Genome-to-Genome Distance calculator [27].

### Genome annotation

The protein annotations produced from PGAP [57] for H6S3^T^, H6W5, H10E4, H19S6^T^ and H4N4^T^ were queried against the PLant-associated BActeria web resource (PLaBAse) database using the DIAMOND MEGAN pipeline [58]. First, the PLaBAse PGPT-db from 01/02/2022 was downloaded and used to build a database in DIAMOND v2.0.11.149 [59]. Each annotated protein file was compared to the database using the BlastP command. To identify high sequence identity alignments between the genomes and the PGPT-db, a query cut-off of 97% and percentage identity equal or greater to than 50 were used. These cut-offs were originally designed for high sequence identify alignments of virulence genes against virulence factors within the same pipeline [60]. The alignments output was then entered into the MEGAN pipeline and mapped against the corresponding mgPGPT-mapping-db in MEGAN version 6.24.0. community edition [61].

Krona plots were created to visualise the PGPT genes identified as groupings defined by their interaction with plants (direct/indirect) and further specific roles [62]. The annotated protein sequences were uploaded to the PGPT-pred online tool (available https://plabase.informatik.uni-tuebingen.de/pb/form.php?var=PGPT-Pred) and queried against the BlastP+HMMER Aligner/Mapper. Finally, to determine if novel isolates are plant-associated bacteria the PIFAR-BASE was used to identify ‘plant bacterial only interaction factors’ from the annotated protein files for each isolate using the BlastP+HMMER Aligner/Mapper.

To further understand the potential of these bacteria as pathogens further comparisons were made against the Virulence Factor Database [63] downloaded on the 26th of July 2022. Genome sequences were queried in DIAMOND by the BlastP command with the same sequence identities and cut-off values as specified for the PGPT database.

### Physiology and chemotaxonomy

#### Morphology of cells and colonies

Light microscopy was used to assess cell length and width, as well as strain motility and morphology. An Olympus SC180 camera linked with CellSens v1.11 microscopy imaging software was used to record all results (Olympus Life Science, Tokyo, Japan). Negative staining of isolates followed by transmission electron microscopy (FEI Tecnai 12,120 kV BioTwin Spirit TEM) was used to observe the flagella arrangement. Negative staining was performed as previously published [64]. The morphology for colonies of all strains was assessed on tryptone soy agar (TSA, Sigma) incubated at 28 °C for 48 h.

#### Growth characteristics

The full range of temperatures at which growth was assessed was 4, 10, 25, 28, 30, 37 and 41 °C on TSA from 24 h to 7 days. To test the pH survival range, the pH of tryptone soy broth (TSB, Sigma) was adjusted using 1 M sodium acetate/acetic acid and 5 M carbonate/bicarbonate buffers to create a set of broths ranging from 4 to 10 pH in increments of 1. Survival in a range of salts concentrations from 1 to 7%, in increments of 1%, was tested by the addition of 1% w/v NaCl to saline-free nutrient broth (3 g l¯^1^ beef extract, 5 g l^− 1^ peptone). Both pH and salt tolerance broths were inoculated in triplicate with individual colonies for each strain and incubated overnight at 30 °C, shaking at 180 RPM.

#### Antibiotic testing

Antibiotic resistance against penicillin V 10 μg, penicillin G 10 μg, tetracycline 30 μg, ampicillin 10 μg, chloramphenicol 30 μg, colistin sulphate 10 μg, streptomycin 25 μg, cefotaxime 5 μg, ciprofloxacin 10 μg, cefepime 30 μg, gentamycin 10 μg and kanamycin 30 μg was tested. Mid-log range bacterial lawns were made on TSA by spread-plating 100 μL of mid-log phase overnight culture and six antibiotic discs were applied at equal distances using a disc dispenser (Oxoid). Plates were incubated at 30 °C for 24 h after which the zone of clearance was checked to determine if the strains were sensitive. Resistance was concluded in no zone of clearance was recorded. Included in all tests were the type strain of *L. adecarboxylata* LMG 2803^T^ and LMG 2650, a strain of *L. adecarboxylata* isolated from *Mangifera indica* (mango).

#### Substrate utilisation and enzyme activity

Phenotypic tests were performed using the commercial assays API 20E, API50CHB/E, API 32 (bioMérieux) and GEN III GN/GP microplates (Biolog) which were used according to the manufacturer’s instructions. The strains tested were H10E4, H20N5, H6S3^T^, H6W8, H6W5, H6S9, H4N4^T^, H18E8 and H19S6^T^ which covered a range of strains from each of the four clusters shown in the MLSA phylogenetic tree. All API galleries were read after 24 h incubation at 37 °C with the API 50 CH/B galleries read again at 48 h. The GEN III microplates were incubated at 30 °C and scored at 16 h and again at 24 h before false positives could occur. Both the type strain of *L. adecarboxylata* LMG 2803^T^ and LMG 2650, the strain isolated from mango were included as reference strains. Oxidase and catalase activity were tested using Kovács reagent (1% tetramethyl-p-phenylenediamine dihydrochloride) and 3% v/v H_2_O_2_, respectively.

#### Fatty acid methyl ester profiles

Fatty Acid Methyl Ester (FAME) profiles were determined for strains LMG 2803^T^ and H10E4 (Cluster 1); H6S3^T^, H6S9, H6W5 andH20N5 (Cluster 2); H4N4^T^ (Cluster 3) and H19S6^T^ (Cluster 4). FERA Science Ltd. performed the service after strains were grown on TSA at 30 °C for 24 h. The Sherlock Microbial Identification System Version 6.4 (MIDI Inc.) protocol was followed, and results were compared against the RTSBA6 6.21 library.

## Supplementary Information

**Additional file 1: Table S1.** List of strains included in this study along with location, year of isolation, source and GenBank accession numbers for MLSA sequences. **Table S2.** Genome features of the strains sequenced in this study including accession numbers, size, G + C content etc. **Table S3.** Average amino acid identity (AAI) values between *Leclercia* and *Silvania* species. **Table S4.** Positive phenotypic characteristics shared by members of the genera *Leclercia* and *Silvania.* **Table S5.** Alternative MLSA sequencing primers used in this study.

**Additional file 2: Fig. S1.** ML phylogenetic tree based on 16S rRNA gene sequences for novel species and genus described in this study as well as the closest phylogenetic relatives. **Fig. S2.** ERIC PCR patterns generated for strains of *Leclercia adecarboxylata*, *Leclercia tamurae* sp. nov., *Silvania hatchlandensis* gen. nov. sp. nov. and *Silvania confinis* sp. nov. **Fig. S3.** TEM images of *Leclercia tamurae* sp. nov. H6S3^T^, *Silvania hatchlandensis* gen. nov. sp. nov. H19S6^T^ and *Silvania confinis* sp. nov. H4N4^T^.

## Data Availability

The sequence data generated and analysed in this study are available at NCBI (https://www.ncbi.nlm.nih.gov/) under the following accession numbers: OM987253 – OM987254 and OM987255 – OM987256 (16S rRNA gene); ON529792 – ON529803 (*atpD*); ON529804 – ON529815 (*gyrB*); ON529816 – ON529827 (*infB*); ON529828 – ON529839 (*rpoB*); JAMGZJ000000000 – JAMGZK000000000 (*Silvania* whole genome sequences) and JAMHKR000000000 – JAMHKT000000000 (*Leclercia* whole genome sequences).

## Abbreviations

AOD: Acute oak Decline
MLSA: Multilocus Sequencing Analysis
ANI: Average Nucleotide Identity
dDDH: digitial DNA-DNA Hybridisation
AAI: Average Amino Identity
PGPB: Plant Growth Promoting Bacteria
PGPT: Plant Growth Promoting Trait
PLaBAse: PLant-associated BActeria web resource
PIFAR: Plant Bacterial Only Interaction Factors
VFDB: Virulence Factor Database
T6SS: Type VI Secretion System
TSA: Tryptone Soy Agar
LAMP: Loop Mediated Isothermal Amplification
EE: Enterobacteriaceae Enrichment
EMB: Eosin Methyl Blue
LB: Luria-Bertani
NA: Nutrient agar
GBDP: Genome Blast Distance Phylogeny
SPR: Subtree Pruning and Regrafting
TSB: Tryptone Soy Broth

## References

[1] O'Hara CM, Farmer J. III Leclercia. In Bergey's Man Syst Archaea Bact. (eds Trujillo, ME, Dedysh S, De Vos P, Hedlund B, Kampfer P, Rainey FA, Whitman WB). 2015; 1-15.

[2] Leclerc H (1962). Étude biochemique d’ Enterobacteriaceae pigmentées. Ann Inst Pasteur.

[3] Ewing WH, Fife MA (1972). Enterobacter agglomerans (Beijerinck) comb. nov. (the Herbicola-Lathyri Bacteria). Int J Syst Bacteriol.

[4] Brenner DJ, Starr M, Stolp H, Trüper HG, Schlegel HG (1981). Introduction to the family Enterobacteriaceae. The prokaryotes: a handbook on habitats, isolation, and identification of bacteria.

[5] Farmer JJ, Davis BR, Hickman-Brenner FW, McWhorter A, Huntley-Carter GP, Asbury MA (1985). Biochemical identification of new species and biogroups of Enterobacteriaceae isolated from clinical specimens. J Clin Microbiol.

[6] Tamura K, Sakazaki R, Kosako Y, Yoshizaki E (1986). Leclercia adecarboxylata gen. nov., comb. nov., formerly known as Escherichia adecarboxylata Kazumichi. Curr Microbiol.

[7] Shin GW, You MJ, Lee HS, Lee CS (2012). Catheter-related bacteremia caused by multidrug-resistant Leclercia adecarboxylata in a patient with breast cancer. J Clin Microbiol.

[8] Eiland EH, Siddiqui H, Goode AM, Leeth SD (2013). Pneumonia due to multidrug-resistant *Leclercia adecarboxylata*. Am J Heal Pharm.

[9] Choudhary M, Choudhary BK, Bhoyar S, Kale SB, Chaudhari SP, Bera BC (2018). Isolation and characterization of multidrug-resistant Leclercia species from animal clinical case. Lett Appl Microbiol.

[10] Keren Y, Keshet D, Eidelman M, Geffen Y, Raz-Pasteur A, Hussein K (2014). Is Leclercia adecarboxylata a new and unfamiliar marine pathogen?. J Clin Microbiol.

[11] Tie Y, Zhu W, Zhang C, Yin L, Zhang Y, Liu L (2021). Identification of two Myrosinases from a Leclercia adecarboxylata strain and investigation of its tolerance mechanism to Glucosinolate hydrolysate. J Agric Food Chem.

[12] Hönemann M, Viehweger A, Dietze N, Johnke J, Rodloff AC (2022). Leclercia pneumoniae sp. nov., a bacterium isolated from clinical specimen in Leipzig, Germany. Int J Syst Evol Microbiol.

[13] Moradi-Amirabad Y, Rahimian H, Babaeizad V, Denman S (2019). Brenneria spp. and Rahnella victoriana associated with acute oak decline symptoms on oak and hornbeam in Iran. For Pathol.

[14] Ruffner B, Schneider S, Meyer J, Queloz V, Rigling D (2020). First report of acute oak decline disease of native and non-native oaks in Switzerland. New Dis Reports.

[15] Duarte CFL, PNE S (2022). First report of Brenneria goodwinii causing acute oak decline on *Quercus suber* in Portugal. J. Plant Pathol..

[16] Tkaczyk M, Celma L, Ruņģis DE, Bokuma G. First report of Brenneria goodwinii and Gibbsiella quercinecans bacteria, detected on weaken oak trees in Poland. Balt For. 2021;27.

[17] Zalkalns O, Celma L. The distribution of bacteria Gibbsiella quercinecans and Brenneria goodwinii in oak (Quercus robur L.) stands in Latvia. IOP Conf Ser earth. Environ Sci. 2021;875.

[18] Denman S, Doonan J, Ransom-Jones E, Broberg M, Plummer S, Kirk S (2018). Microbiome and infectivity studies reveal complex polyspecies tree disease in acute oak decline. ISME J..

[19] Sinclair WA, Hudler GW (1988). Tree Delince: four concepts of causality. J Arboric.

[20] Denman S, Brown N, Vanguelova E, Crampton B. Temperate oak declines: biotic and abiotic predisposition drivers in Forest Microbiology (eds Asiegby, FO, Kovalchuk, A). Academic Press; 2022. p. 239-63.

[21] Philippot L, Raaijmakers JM, Lemanceau P, Van Der Putten WH (2013). Going back to the roots: the microbial ecology of the rhizosphere. Nat Rev Microbiol.

[22] Pinho D, Barroso C, Froufe H, Brown N, Vanguelova E, Egas C (2020). Linking tree health, rhizosphere physicochemical properties, and microbiome in acute oak decline. Forests..

[23] Scarlett K, Denman S, Clark DR, Forster J, Vanguelova E, Brown N (2021). Relationships between nitrogen cycling microbial community abundance and composition reveal the indirect effect of soil pH on oak decline. ISME J.

[24] Sun Q, Wang H, Shu L, Dong N, Yang F, Zhou H, et al. *Leclercia adecarboxylata* From Human Gut Flora Carries mcr-4.3 and blaIMP-4-Bearing Plasmids. Front Microbiol. 2019;10. 10.3389/fmicb.2019.02805 PMC6906143 31866974

[25] Xu YY, Huang CJ, Xu L, Jiang XW, Xu XW, Xu XW (2020). Complete genome sequences of Leclercia sp. W6 and W17 isolated from a gastric Cancer patient. Curr Microbiol.

[26] Naum M, Brown EW, Mason-Gamer RJ (2008). Is 16S rDNA a reliable phylogenetic marker to characterize relationships below the family level in the enterobacteriaceae?. J Mol Evol.

[27] Goris J, Konstantinidis KT, Klappenbach JA, Coenye T, Vandamme P, Tiedje JM (2007). DNA-DNA hybridization values and their relationship to whole-genome sequence similarities. Int J Syst Evol Microbiol.

[28] Konstantinidis KT, Tiedje JM (2005). Towards a genome-based taxonomy for prokaryotes. J Bacteriol.

[29] Konstantinidis KT, Tiedje JM (2005). Genomic insights that advance the species definition for prokaryotes. Proc Natl Acad Sci U S A.

[30] Luo C, Rodriguez-R LM, Konstantinidis KT (2014). MyTaxa: an advanced taxonomic classifier for genomic and metagenomic sequences. Nucleic Acids Res.

[31] Nicholson AC, Gulvik CA, Whitney AM, Humrighouse BW, Bell ME, Holmes B (2020). Division of the genus chryseobacterium: observation of discontinuities in amino acid identity values, a possible consequence of major extinction events, guides transfer of nine species to the genus epilithonimonas, eleven species to the genus kaistella, a. Int J Syst Evol Microbiol.

[32] Soutar CD, Stavrinides J (2022). Phylogenomic analysis of the Erwiniaceae supports reclassification of Kalamiella piersonii to Pantoea piersonii comb. nov. and Erwinia gerundensis to the new genus Duffyella gen. Nov. as Duffyella gerundensis comb. nov. Mol Gen Genomics.

[33] Brown N, Vanguelova E, Parnell S, Broadmeadow S, Denman S (2018). Predisposition of forests to biotic disturbance: predicting the distribution of acute oak decline using environmental factors. For Ecol Manag.

[34] Kang SM, Shahzad R, Khan MA, Hasnain Z, Lee KE, Park HS (2021). Ameliorative effect of indole-3-acetic acid- and siderophore-producing Leclercia adecarboxylata MO1 on cucumber plants under zinc stress. J Plant Interact.

[35] Toth IK, Pritchard L, Birch PRJ (2006). Comparative genomics reveals what makes an Enterobacterial plant pathogen. Annu Rev Phytopathol.

[36] Monteiro RA, Balsanelli E, Wassem R, Marin AM, Brusamarello-Santos LCC, Schmidt MA (2012). Herbaspirillum-plant interactions: Microscopical, histological and molecular aspects. Plant Soil.

[37] De Baere T, Wauters G, Huylenbroeck A, Claeys G, Peleman R, Verschraegen G (2001). Isolations of leclercia adecarboxylata from a patient with a chronically inflamed gallbladder and from a patient with sepsis without focus. J Clin Microbiol.

[38] El J, GDW C, Curtis R, Baird M (2003). Enterobacteriaceae enrichment (EE) broth. Prog Ind Microbiol.

[39] Niemann S, Pühler A, Tichy HV, Simon R, Selbitschka W (1997). Evaluation of the resolving power of three different DNA fingerprinting methods to discriminate among isolates of a natural rhizobium meliloti population. J Appl Microbiol.

[40] Brady C, Cleenwerck I, Venter S, Vancanneyt M, Swings J, Coutinho T (2008). Phylogeny and identification of Pantoea species associated with plants, humans and the natural environment based on multilocus sequence analysis (MLSA). Syst Appl Microbiol.

[41] Coenye T, Falsen E, Vancanneyt M, Hoste B, Govan JRW, Kersters K (1999). Classification of Alcaligenes faecalis-like isolates from the environment and human clinical samples as Ralstonia gilardii sp. nov. Int J Syst Bacteriol.

[42] Okonechnikov K, Golosova O, Fursov M, Varlamov A, Vaskin Y, Efremov I (2012). Unipro UGENE: a unified bioinformatics toolkit. Bioinformatics..

[43] Benson DA, Cavanaugh M, Clark K, Karsch-Mizrachi I, Lipman DJ, Ostell J (2013). GenBank. Nucleic Acids Res.

[44] Tamura K, Stecher G, Kumar S (2021). MEGA11: molecular evolutionary genetics analysis version 11. Mol Biol Evol.

[45] Yoon SH, Ha SM, Kwon S, Lim J, Kim Y, Seo H (2017). Introducing EzBioCloud: a taxonomically united database of 16S rRNA gene sequences and whole-genome assemblies. Int J Syst Evol Microbiol.

[46] Lefort V, Longueville J-E, Gascuel O (2017). SMS: smart model selection in PhyML. Mol Biol Evol.

[47] Guindon S, Gascuel O (2003). A simple, fast, and accurate algorithm to estimate large phylogenies by maximum likelihood. Syst Biol.

[48] Versalovic J, Koeuth T, Lupski R (1991). Distribution of repetitive DNA sequences in eubacteria and application to finerpriting of bacterial enomes. Nucleic Acids Res.

[49] Bolger AM, Lohse M, Usadel B (2014). Trimmomatic: a flexible trimmer for Illumina sequence data. Bioinformatics..

[50] Bankevich A, Nurk S, Antipov D, Gurevich AA, Dvorkin M, Kulikov AS (2012). SPAdes: a new genome assembly algorithm and its applications to single-cell sequencing. J Comput Biol a J Comput Mol cell Biol.

[51] Meier-Kolthoff JP, Göker M (2019). TYGS is an automated high-throughput platform for state-of-the-art genome-based taxonomy. Nat Commun.

[52] Meier-Kolthoff JP, Auch AF, Klenk H-P, Göker M (2013). Genome sequence-based species delimitation with confidence intervals and improved distance functions. BMC Bioinformatics..

[53] Farris JS (1972). Estimating Phlogenetic Trees from Distance Matrices. Am. Nat..

[54] Lefort V, Desper R, Gascuel O (2015). FastME 2.0: a comprehensive, accurate, and fast distance-based phylogeny inference program. Mol Biol Evol.

[55] Jain C, Rodriguez-R LM, Phillippy AM, Konstantinidis KT, Aluru S (2018). High throughput ANI analysis of 90K prokaryotic genomes reveals clear species boundaries. Nat Commun.

[56] Rodriguez-R LM, Konstantinidis KT (2016). The enveomics collection: a toolbox for specialized analyses of microbial genomes and metagenomes. PeerJ Prepr.

[57] Tatusova T, DiCuccio M, Badretdin A, Chetvernin V, Nawrocki EP, Zaslavsky L (2016). NCBI prokaryotic genome annotation pipeline. Nucleic Acids Res.

[58] Bağcı C, Patz S, Huson DH (2021). DIAMOND+MEGAN: fast and easy taxonomic and functional analysis of short and long microbiome sequences. Curr Protoc.

[59] Buchfink B, Reuter K, Drost HG (2021). Sensitive protein alignments at tree-of-life scale using DIAMOND. Nat Methods.

[60] Doonan J, Denman S, Pachebat JA, McDonald JE (2019). Genomic analysis of bacteria in the acute oak decline pathobiome. Microb Genomics.

[61] Huson DH, Beier S, Flade I, Górska A, El-Hadidi M, Mitra S (2016). MEGAN Community edition-interactive exploration and analysis of large-scale microbiome sequencing data. PLoS Comput Biol.

[62] Ondov BD, Bergman NH, Phillippy AM (2011). Interactive metagenomic visualization in a web browser. BMC Bioinformatics.

[63] Liu B, Zheng D, Zhou S, Chen L, Yang J (2022). VFDB 2022: a general classification scheme for bacterial virulence factors. Nucleic Acids Res.

[64] Brady C, Asselin JA, Beer S, Brurberg MB, Crampton B, Venter S (2022). Rahnella perminowiae sp. nov., Rahnella bonaserana sp. nov., Rahnella rivi sp. nov. and Rahnella ecdela sp. nov., isolated from diverse environmental sources, and emended description of the genus Rahnella. Int J Syst Evol Microbiol.

