# Supplementary material for: Description of a novel species of Leclercia, Leclercia tamurae sp. nov. and proposal of a novel genus Silvania gen. nov. containing two novel species Silvania hatchlandensis sp. nov. and Silvania confinis sp. nov. isolated from the rhizosphere of oak
Source: BMC Microbiol. 2022 Dec 2;22:289. doi: 10.1186/s12866-022-02711-x (PMC9716735; doi:10.1186/s12866-022-02711-x)
Supplement: Supplementary file 1 — Additional file 1: Table S1. List of strains included in this study along with location, year of isolation, source and GenBank accession numbers for MLSA sequences. Table S2. Genome features of the strains sequenced in this study including accession numbers, size, G + C content etc. Table S3. Average amino acid identity (AAI) values between Leclercia and Silvania species. Table S4. Positive phenotypic characteristics shared by members of the genera Leclercia and Silvania. Table S5. Alternative MLSA sequencing primers used in this study. [file 12866_2022_2711_MOESM1_ESM.docx]

**Table S1**: Strains investigated in this study.

|  | **GenBank accession numbers** | | | |  |  |  |  |
| --- | --- | --- | --- | --- | --- | --- | --- | --- |
| **Strain** | **Location** | **Year of isolation** | **Source** | **Clonal strains** | ***atpD*** | ***gyrB*** | ***infB*** | ***rpoB*** |
| ***Leclercia adecarboxylata*** |  |  |  |  |  |  |  |  |
| H10E4 | Hatchlands, Guildford, UK | 2020 | *Quercus robur* (healthy) rhizosphere soil | None | ON529800 | ON529812 | ON529824 | ON529836 |
| H10E8 | Hatchlands, Guildford, UK | 2020 | *Quercus robur* (healthy) rhizosphere soil | None | ON529798 | ON529810 | ON529822 | ON529834 |
| H9E1a | Hatchlands, Guildford, UK | 2020 | *Quercus robur* (AOD) rhizosphere soil | None | ON529799 | ON529811 | ON529823 | ON529835 |
| ***Leclercia tamurae* sp. nov.** |  |  |  |  |  |  |  |  |
| H6S3^T^ | Hatchlands, Guildford, UK | 2020 | *Quercus robur* (healthy) rhizosphere soil | H6W8 | ON529795 | ON529807 | ON529819 | ON529831 |
| H6W5 | Hatchlands, Guildford, UK | 2020 | *Quercus robur* (healthy) rhizosphere soil | H6S9 | ON529797 | ON529809 | ON529821 | ON529833 |
| H6W8 | Hatchlands, Guildford, UK | 2020 | *Quercus robur* (healthy) rhizosphere soil | H6S3^T^ | ON529792 | ON529804 | ON529816 | ON529828 |
| H6W6a | Hatchlands, Guildford, UK | 2020 | *Quercus robur* (healthy) rhizosphere soil | H20N5 | ON529793 | ON529805 | ON529817 | ON529829 |
| H20N5 | Hatchlands, Guildford, UK | 2020 | *Quercus petraea* (healthy) rhizosphere soil | H6W6a | ON529794 | ON529806 | ON529818 | ON529830 |
| H6S9 | Hatchlands, Guildford, UK | 2020 | *Quercus robur* (healthy) rhizosphere soil | H6W5 | ON529796 | ON529808 | ON529820 | ON529832 |
| ***Silvania hatchlandensis* sp. nov.** |  |  |  |  |  |  |  |  |
| H19S6^T^ | Hatchlands, Guildford, UK | 2020 | *Quercus petraea* (AOD) rhizosphere soil | None | ON529803 | ON529815 | ON529827 | ON529839 |
| H18E8 | Hatchlands, Guildford, UK | 2020 | *Quercus robur* (healthy) rhizosphere soil | None | ON529802 | ON529814 | ON529826 | ON529838 |
| ***Silvania confinis* sp. nov.** |  |  |  |  |  |  |  |  |
| H4N4^T^ | Hatchlands, Guildford, UK | 2020 | *Quercus robur* (healthy) rhizosphere soil | None | ON529801 | ON529813 | ON529825 | ON529837 |

**Table S2:** Whole Genome sequence information of strains investigated in this study.

| **Strain** | **GenBank**  **accession** | **Biosample number** | **Size (Mbp)** | **Number of contigs** | **N50** | **Number of coding sequences** | **Numbers of RNAs** | **GC content (mol %)** |
| --- | --- | --- | --- | --- | --- | --- | --- | --- |
| ***Leclercia adecarboxylata*** |  |  |  |  |  |  |  |  |
| H10E4 | JAMHKT000000000 | SAMN28207097 | 4.83 | 61 | 336 719 | 4 585 | 91 | 55.6 |
| ***Leclercia tamurae*** |  |  |  |  |  |  |  |  |
| H6S3^T^ | JAMHKS000000000 | SAMN28207096 | 4.71 | 80 | 268 955 | 4 465 | 96 | 56.4 |
| H6W5 | JAMHKR000000000 | SAMN28207095 | 4.86 | 94 | 323 472 | 4 647 | 94 | 56.4 |
| ***Silvania hatchlandensis*** |  |  |  |  |  |  |  |  |
| H19S6^T^ | JAMGZK000000000 | SAMN28207118 | 4.78 | 67 | 207 460 | 4 493 | 88 | 55.9 |
| ***Silvania confinis*** |  |  |  |  |  |  |  |  |
| H4N4^T^ | JAMGZJ000000000 | SAMN28207119 | 4.87 | 82 | 225 593 | 4 675 | 92 | 55.7 |

**Table S3:** Average amino acid identity (AAI) values between *Leclercia* and *Silvania* species. Shaded boxes represent alignments which exceed the recommended species delimitation value of 96 %.

|  | **1** | **2** | **3** | **4** | **5** | **6** | **7** | **8** | **9** | **10** | **11** | **12** | **13** | **14** | **15** | **16** | **17** |  |
| --- | --- | --- | --- | --- | --- | --- | --- | --- | --- | --- | --- | --- | --- | --- | --- | --- | --- | --- |
| **1** | 100 | 99 | 99 | 99 | 99 | 99 | 89 | 95 | 95 | 95 | 95 | 94 | 93 | 93 | 93 | 91 | 90 | |
| **2** | 99 | 100 | 99 | 98 | 99 | 98 | 89 | 95 | 95 | 94 | 95 | 93 | 93 | 93 | 93 | 91 | 90 | |
| **3** | 99 | 99 | 100 | 99 | 99 | 98 | 89 | 95 | 95 | 95 | 95 | 93 | 93 | 93 | 93 | 91 | 90 | |
| **4** | 99 | 98 | 99 | 100 | 98 | 98 | 89 | 95 | 94 | 94 | 94 | 93 | 92 | 93 | 93 | 91 | 90 | |
| **5** | 99 | 99 | 99 | 98 | 100 | 98 | 89 | 95 | 95 | 94 | 94 | 93 | 92 | 93 | 94 | 91 | 90 | |
| **6** | 99 | 98 | 98 | 98 | 98 | 100 | 88 | 94 | 94 | 93 | 94 | 93 | 92 | 93 | 93 | 91 | 90 | |
| **7** | 89 | 89 | 89 | 89 | 89 | 88 | 100 | 89 | 89 | 88 | 89 | 89 | 89 | 89 | 88 | 88 | 88 | |
| **8** | 95 | 95 | 95 | 95 | 95 | 94 | 89 | 100 | 98 | 95 | 95 | 94 | 93 | 93 | 93 | 91 | 91 | |
| **9** | 95 | 95 | 95 | 94 | 95 | 94 | 89 | 98 | 100 | 95 | 95 | 94 | 93 | 93 | 93 | 91 | 91 | |
| **10** | 95 | 94 | 95 | 94 | 94 | 93 | 88 | 95 | 95 | 100 | 98 | 94 | 93 | 93 | 92 | 91 | 90 | |
| **11** | 95 | 95 | 95 | 94 | 94 | 94 | 89 | 95 | 95 | 98 | 100 | 94 | 93 | 93 | 92 | 91 | 91 | |
| **12** | 94 | 93 | 93 | 93 | 93 | 93 | 89 | 94 | 94 | 94 | 94 | 100 | 99 | 98 | 95 | 91 | 90 | |
| **13** | 93 | 93 | 93 | 92 | 92 | 92 | 89 | 93 | 93 | 93 | 93 | 99 | 100 | 97 | 93 | 90 | 89 | |
| **14** | 93 | 93 | 93 | 93 | 93 | 93 | 89 | 93 | 93 | 93 | 93 | 98 | 97 | 100 | 94 | 90 | 90 | |
| **15** | 93 | 93 | 93 | 93 | 94 | 93 | 88 | 93 | 93 | 92 | 92 | 95 | 93 | 94 | 100 | 91 | 90 | |
| **16** | 91 | 91 | 91 | 91 | 91 | 91 | 88 | 91 | 91 | 91 | 91 | 91 | 90 | 90 | 91 | 100 | 95 | |
| **17** | 90 | 90 | 90 | 90 | 90 | 90 | 88 | 91 | 91 | 90 | 91 | 90 | 89 | 90 | 90 | 95 | 100 | |

1 = *Leclercia adecarboxylata* NBRC 102595^T^ (GCA_001515505), 2 = *Leclercia adecarboxylata* L21 (GCA_011045715), 3 = *Leclercia adecarboxylata* H10E4 (GCA_025566045), 4 = *Leclercia adecarboxylata* 16400 (GCA_014489435), 5 = *Leclercia adecarboxylata* E1 (GCA_008931445), 6 = *Leclercia adecarboxylata* E61 (GCA_008931385), 7 = *Leclercia pneumoniae* 49125^T^ (GCA_018987305), 8 = *Leclercia* *tamurae* H6S3^T^ (GCA_025566055), 9 = *Leclercia* *tamurae* H6W5 (GCA_025566025), 10 = *Leclercia* sp. G3L (GCA_021117075), 11 = *Leclercia* sp. 119287 (GCA_009734485), 12 = *Leclercia* Colony 189 (GCA_018513965), 13 = *Leclercia* sp. LSNIH1 (GCA_002902985), 14 = *Leclercia* sp. W6 (GCA_003336345), 15 = *Leclercia* sp. Z96-1 (GCA_006171285) , 16 = *Silvania hatchlandensis* H19S6^T^ (GCA_025564065), 17 = *Silvania confinis* H4N4^T^ (GCA_025564085).

**Table S4**: Positive phenotypic characteristics shared by members of the genera *Leclercia* and *Silvania*

| **Characteristic** | ***Leclercia adecarboxylata***  **(*n* = 4)** | ***Leclercia tamurae***  **(*n* = 5)** | ***Silvania hatchlandensis* (*n* = 2)** | ***Silvania***  ***confinis***  **H4N4^T^** |
| --- | --- | --- | --- | --- |
| **Acidification of:** |  |  |  |  |
| glucose | + | + | + | + |
| mannitol | + | + | + | + |
| sorbitol |  | + | + | + |
| rhamnose | + | + | v | v |
| melibiose | + | + | + | + |
| amygdalin | + | + | + | + |
| L-arabinose | + | + | + | + |
| D-arabinose |  |  | + | + |
| glycerol | + | + | + | + |
| D-ribose | + | + | + | + |
| D-xylose | + | + | + | + |
| D-galactose | + | + | + | + |
| D-fructose | + | + | + | + |
| D-mannose | + | + | + | + |
| dulcitol |  | + | + | + |
| *N*-acetylglucosamine | + | + | + | + |
| arbutin | + | + | + | + |
| esculin ferric citrate | + | + | + | + |
| salicin | + | + | + | + |
| D-cellobiose | + | + | + | + |
| D-maltose | + | + | + | + |
| D-lactose | + | + | + | + |
| D-trehalose |  | v | + | + |
| D-raffinose |  |  | + | + |
| gentiobiose | + | + | + | + |
| potassium gluconate | + | + | + | + |
| potassium 5-ketogluconate |  | v | + | + |
| **Utilisation of (Biolog):** |  |  |  |  |
| dextrin | + | + |  |  |
| sucrose |  | v | + | + |
| D-salicin | + | v | + | + |
| *N*-acetyl-D-glucosamine | + | + | + | + |
| *N*-acetyl-β-D-mannosamine | + | + | + | + |
| *N*-acetyl neuraminic acid | + | + | + | + |
| 3-methyl glucose |  | v | + | + |
| inosine | + | + | + | + |
| D-glucose-6-phosphate | + | + | + | + |
| D-fructose-6-phosphate | + | + | + | + |
| D-aspartic acid | + |  | + | + |
| glycyl-L-proline | + | + | + | + |
| L-alanine | + | + | + | + |
| L-arginine | + | + | + | + |
| L-aspartic acid | + | + | + | + |
| L-glutamic acid | + | + | + | + |
| L-histidine | + | + | + | + |
| L-serine | + | + | + | + |
| D-galacturonic acid | + | + | + | + |
| L-galactonic acid lactone | + | + | + | + |
| D-gluconic acid | + | + | + | + |
| D-glucuronic acid | + | + | + | + |
| glucuronamide | + | + | + | + |
| mucic acid | + | + | + | + |
| D-saccharic acid | + | + | + | + |
| p-hydroxy-phenylacetic acid | + | + |  |  |
| methyl pyruvate | + | + | + | + |
| D-lactic acid methyl ester | + | + | + | + |
| L-lactic acid | + | + | + | + |
| citric acid |  | v | + | + |
| L-malic acid | + | + | + | + |
| bromo-succinic acid | + | + | + | + |
| tween 40 | + | v | + | + |
| acetoacetic acid | + | + | + | + |
| acetic acid | + | + | + | + |

+, 90 – 100 % strains +; v, variable

**Table S5:** Alternative MLSA sequencing primers used in this study

| **gene** | **Sequence 5’ → 3’** |
| --- | --- |
| **atpD 04.3-R** | CCM AGY ARB GCD GAT ACT TC |
| **infB 03.2-F** | ACG GBA TGR TBA CST TCC TKG |
| **infB 04.2-R** | AGY TTA GAT TTC TGC TGR CG |
