# Supplementary material for: Description of a novel species of Leclercia, Leclercia tamurae sp. nov. and proposal of a novel genus Silvania gen. nov. containing two novel species Silvania hatchlandensis sp. nov. and Silvania confinis sp. nov. isolated from the rhizosphere of oak
Source: BMC Microbiol. 2022 Dec 2;22:289. doi: 10.1186/s12866-022-02711-x (PMC9716735; doi:10.1186/s12866-022-02711-x)
Supplement: Supplementary file 2 — Additional file 2: Fig. S1. ML phylogenetic tree based on 16S rRNA gene sequences for novel species and genus described in this study as well as the closest phylogenetic relatives. Fig. S2. ERIC PCR patterns generated for strains of Leclercia adecarboxylata, Leclercia tamurae sp. nov., Silvania hatchlandensis gen. nov. sp. nov. and Silvania confinis sp. nov. Fig. S3. TEM images of Leclercia tamurae sp. nov. H6S3T, Silvania hatchlandensis gen. nov. sp. nov. H19S6T and Silvania confinis sp. nov. H4N4T. [file 12866_2022_2711_MOESM2_ESM.docx]

Fig. S1 Maximum Likelihood phylogenetic tree based on 16S rRNA gene sequences for species of the novel genus *Silvania* gen. nov., *Leclercia*, the novel species *Leclercia tamurae* sp. nov. and several closest phylogenetic neighbours. Near complete (1,346 bp) 16S rRNA gene sequences were used, with the scale showing the nucleotide substitutions per site and bootstrap values exceeding 50 % from 1000 replicates shown at nodes. Species names are followed by the strain number and GenBank accession number, with ^T^ indicating the type strain. The outgroup is *Plesiomonas shigelloides* NCIMB 9242^T^.


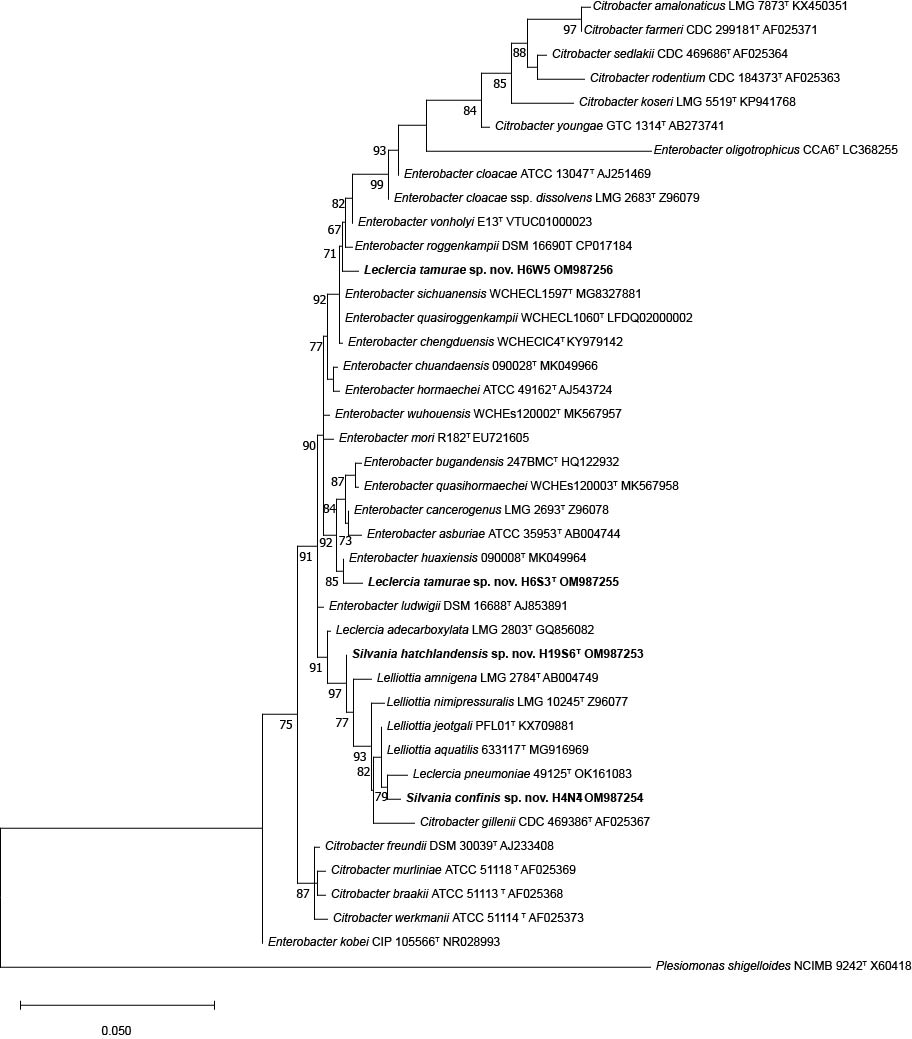


Fig. S2: ERIC PCR fingerprinting patterns generated from strains of *Leclercia adecarboxylata*, *Leclercia tamurae* sp. nov. and the novel genus *Silvania* gen. nov. (1) *Leclercia adecarboxylata* LMG 2803^T^, (2) *Leclercia* *adecarboxylata* LMG 2650, (3) *Leclercia adecarboxylata* H10E4, (4) *Leclercia adecarboxylata* H9E1a, (5) *Leclercia adecarboxylata* H10E8, (6) *Leclercia tamurae* H6S3^T^, (7) *Leclercia tamurae* H6W8, (8) *Leclercia tamurae* H6W6a, (9) *Leclercia tamurae* H20N5, (10) *Leclercia tamurae* H6W5, (11) *Leclercia tamurae* H6S9, (12) *Silvania hatchlandensis* H19S6^T^, (13) *Silvania hatchlandensis* H18E8, (14) *Silvania confinis* H4N4^T^, (15) negative control.

A 1Kb Hyperladder (Bioline) was run as a size marker in both the first and last wells.

All samples were loaded and run on the same agarose gel and the resulting image was cropped to remove negative space only, ensuring that all amplification products are visible.


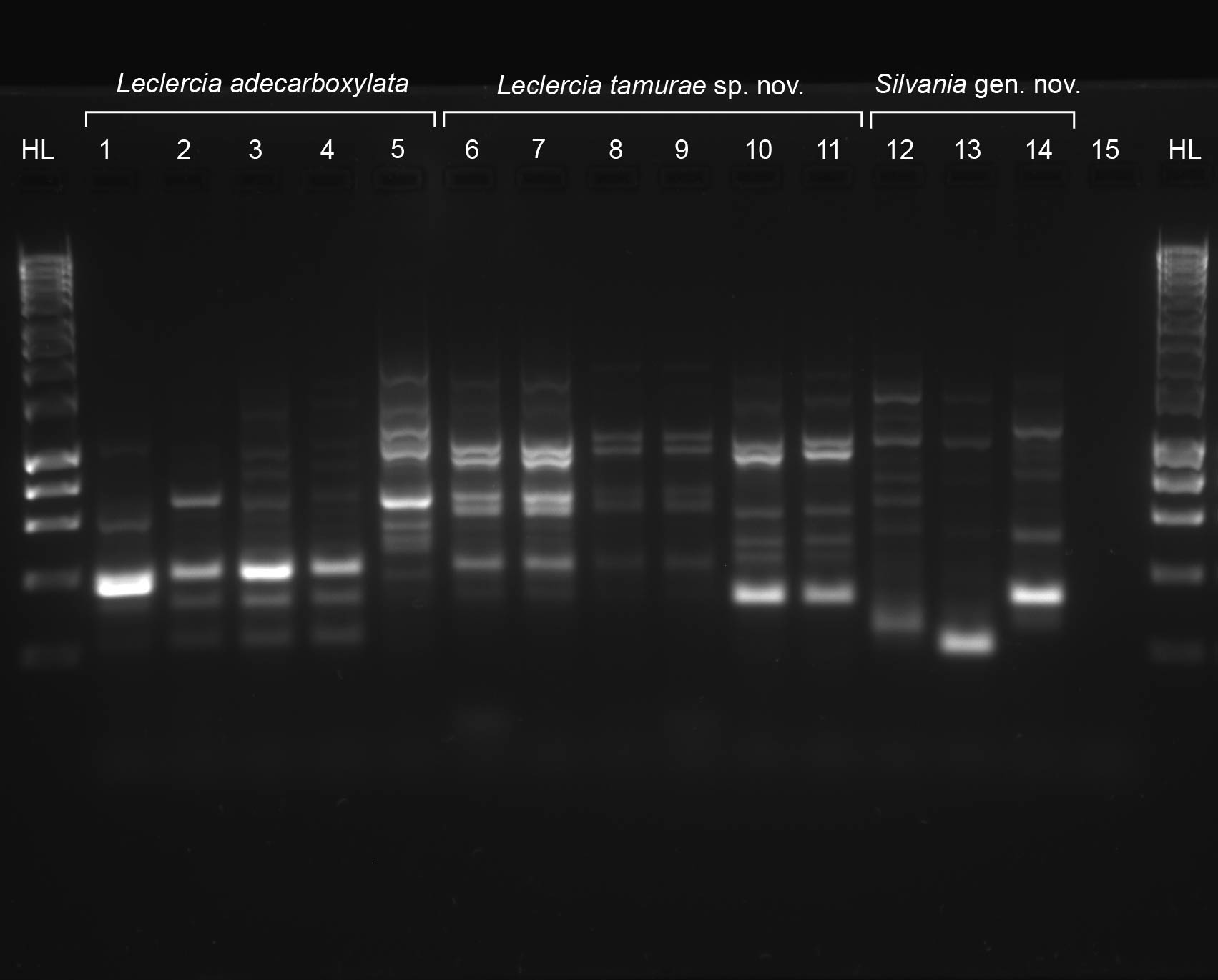


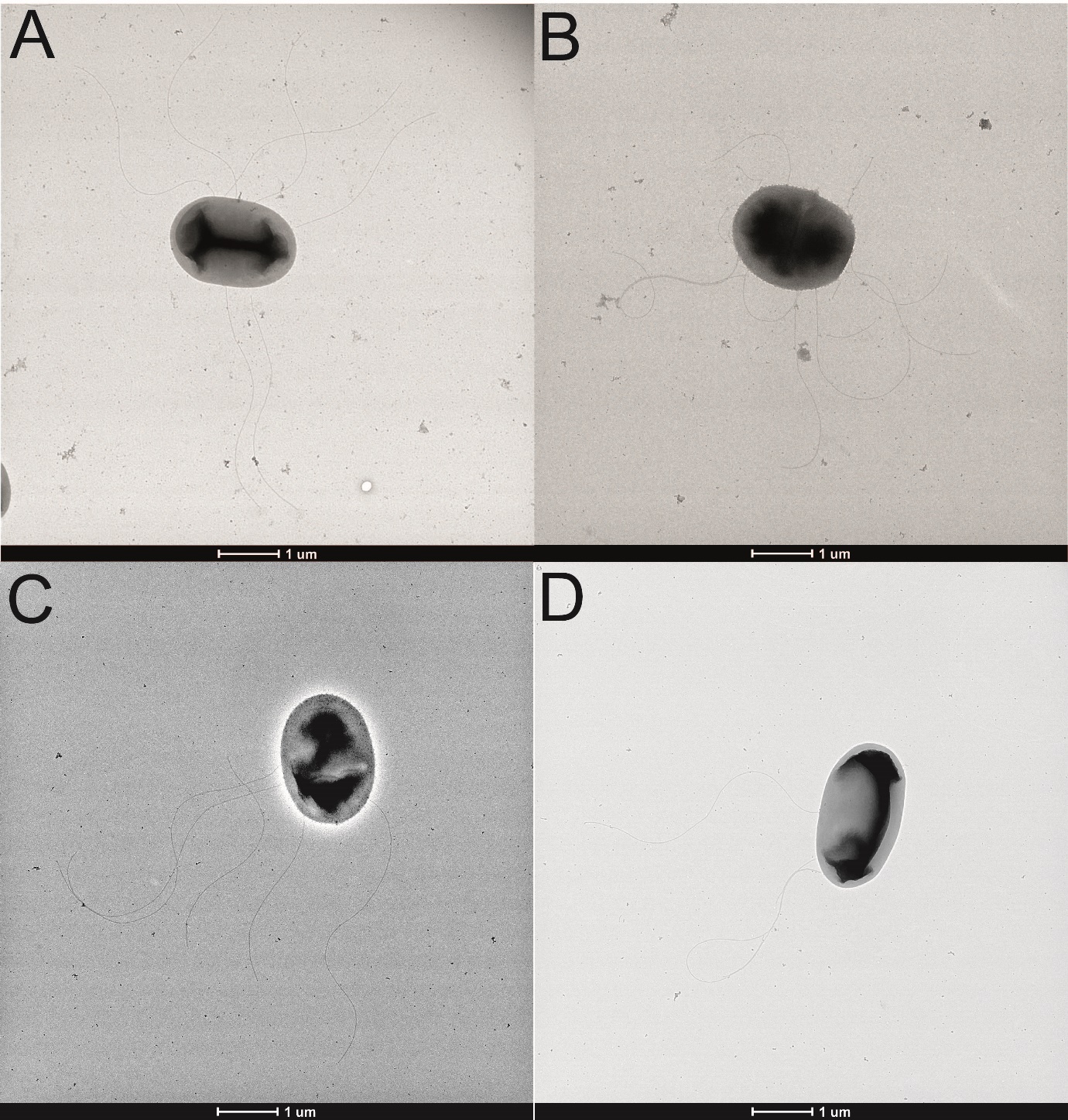
Fig. S3: Transmission electron microscopy images of A) *Leclercia tamurae* H6S3^T^ B) *Leclercia tamurae* H6W5 C) *Silvania hatchlandensis* H19S6^T^ D) *Silvania confinis* H4N4^T^displaying their peritrichous flagella arrangement. Scale bar, 1 µm
